# OBP2A regulates epidermal barrier function and protects against cytotoxic small hydrophobic molecules

**DOI:** 10.1016/j.isci.2024.111093

**Published:** 2024-10-02

**Authors:** Shinobu Nakanishi, Tatsuya Hasegawa, Katsuyuki Maeno, Akira Motoyama, Mitsuhiro Denda

**Affiliations:** 1 Shiseido Global Innovation Center, Yokohama 220-0011, Japan; 2 Institute for Advanced Study of Mathematical Sciences, Meiji University, Nakano-ku, Tokyo 164-8525, Japan

**Keywords:** Dermatology, Physiology, Molecular biology, Cell biology

## Abstract

The skin is constantly exposed to environmental sensory stimuli, which may include harmful volatiles and small hydrophobic molecules. However, the skin’s protective mechanism against the latter agents is unclear. Here, we demonstrate that odorant binding protein 2A (OBP2A) protects epidermal keratinocytes against cytotoxic small hydrophobic molecules. OBP2A is mainly expressed in human epidermal keratinocytes. Cellular resistance to cytotoxic aldehyde and lipids was reduced in keratinocytes when OBP2A was silenced. Furthermore, silencing of OBP2A in a three-dimensional epidermal equivalent model resulted in impairment of epidermal barrier function. Inhibition of OBP2A caused disruption of keratinocyte lipid metabolism and induced endoplasmic reticulum stress. OBP2A expression was markedly decreased in the epidermis of atopic dermatitis lesional skin. In addition, interleukin-13 suppressed the expression of OBP2A in keratinocytes. Overall, our findings suggest that OBP2A regulates epidermal barrier function and contributes to protection of the skin against harmful small hydrophobic molecules.

## Highlights

• Odorant binding protein 2A (OBP2A) is expressed in human epidermal keratinocytes
• OBP2A protects keratinocytes against cytotoxic aldehyde and fatty acid
• OBP2A is involved in lipid metabolism and influences epidermal barrier formation
• OBP2A expression level is decreased in atopic dermatitis lesional skin

## Graphical abstract

**Figure:**
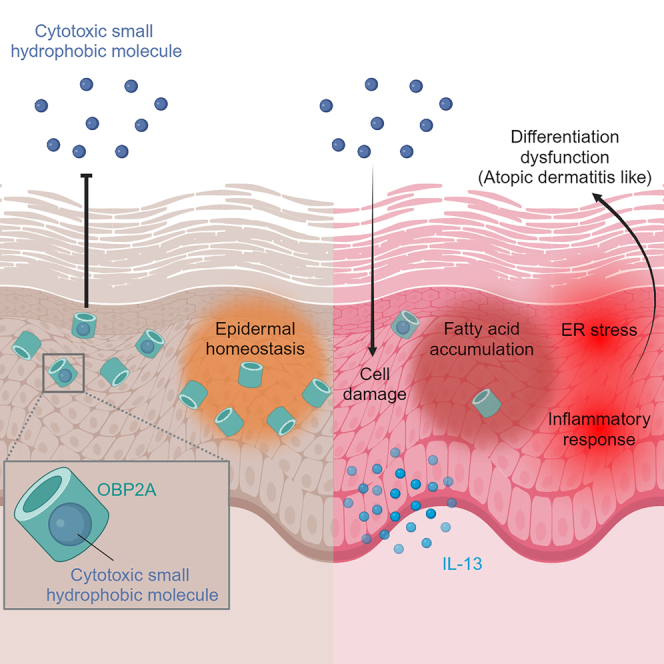

## Introduction

The skin is the largest human organ, and is constantly exposed to various environmental factors and therefore the epidermis, which forms its outermost layer, serves as a barrier against physical, chemical, microbial, and immunological stimuli to maintain homeostasis.^1^^,^^2^^,^^3^^,^^4^ Furthermore, epidermal keratinocytes contain sensory systems that can detect a variety of environmental factors, including volatiles and low-molecular-weight compounds, and can integrate multiple environmental cues to regulate skin homeostasis.^5^ For example, olfactory receptors (ORs) in keratinocytes sense odorants and play important roles in wound healing, differentiation, and keratinization.^6^^,^^7^^,^^8^ In contrast, *trans*-2-nonenal (NE), an aging-related odor compound, decreases the viability of keratinocytes and impairs three-dimensional epidermal construction.^9^ Aldehydes and aliphatic hydrocarbons are thought to cause so-called “sick building” syndrome, as well as skin dryness and itching.^10^^,^^11^ High sensitivity to chemical compounds that further aggravate symptoms is well-known in allergic dermatitis.^12^ However, the nature of the skin’s defense mechanism against harmful volatiles and low-molecular-weight compounds remains unclear.

Mechanistic studies in the nose indicate that not only ORs but also olfactory accessory proteins have crucial roles in olfaction.^13^ Olfactory cells must be protected from harmful volatiles to maintain efficient odorant detection. Odorant binding proteins (OBPs) are among the olfactory accessory proteins that are abundantly expressed in nasal mucosa.^14^^,^^15^ OBPs belong to the lipocalin superfamily and bind with various small hydrophobic molecules. They can be considered as scavengers that protect olfactory cells from toxic volatiles, as well as transporters of odorants and pheromones within the aqueous mucus.^13^^,^^16^ OBPs have been detected not only in nasal mucosa, but also in various organs such as the lung, sweat gland, mammary gland, placenta, ovary, testis, and prostate.^17^^,^^18^ In insects, OBPs are involved in oocyte maturation, larval development, formation of eggshell, gills, and wings, and vision.^19^^,^^20^^,^^21^^,^^22^

We hypothesized that OBPs in human keratinocytes are related to epidermal defense mechanisms against harmful small hydrophobic molecules, and epidermal differentiation.

## Results

### OBP2A is expressed in human epidermal keratinocytes

**Figure 1:**
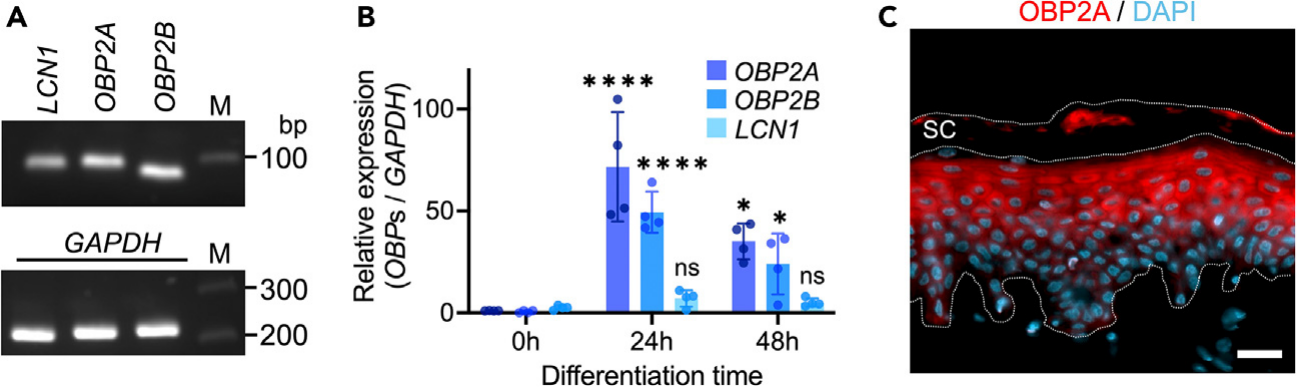
Expression of OBPs in human epidermal keratinocytes (A) qPCR analysis of OBPs in keratinocytes. *GAPDH* was used for RNA quality control. M: Marker. (B) Comparison of relative expression of OBPs (*n* = 4). Anova F value = 18.73, *p* < 0.0006 for *OBP2A*, Anova F value = 22.01, *p* < 0.0003 for *OBP2B*, Anova F value = 2.861, *p* < 0.109 for *LCN1*. (C) Immunofluorescence staining of OBP2A in human skin. Cell nuclei appear in blue. Bars = 20 μm. SC: stratum corneum. Bars and lines represent mean ± SD. ∗: *p* < 0.05, ∗∗∗∗: *p* < 0.0001, ns: not significant in ANOVA with Scheffé’s method. See also Figure S1.

To examine the expression of OBPs in human epidermal keratinocytes, we performed quantitative PCR (qPCR) analysis. Three kinds of OBPs (OBP2A, OBP2B, and LCN1) have been identified in humans,^18^ and the corresponding mRNA transcripts were detected in keratinocytes (Figure 1A). Transcription of *OBP2A* and *OBP2B*, but not *LCN1*, increased significantly with differentiation (Figure 1B). *OBP2A*, *OBP2B*, and *LCN1* mRNAs were also detected in a three-dimensional epidermal equivalent model (3DE-model) (Figure S1A). Because transcription of OBP2A and OBP2B were much higher than LCN1 in differentiated keratinocytes, we determined the protein expression levels of OBP2A and OBP2B by means of enzyme-linked immunosorbent assay (ELISA). OBP2A was detected in both differentiated keratinocytes and the 3DE-model at the protein level (Figure S1B), but OBP2B was below the detection limit of the ELISA system. Immunostaining of human skin tissue samples revealed that OBP2A was mainly expressed by keratinocytes in the upper layer (Figure 1C). Therefore, we focused on OBP2A in subsequent experiments.

### OBP2A protects keratinocytes against cytotoxic aldehyde and fatty acid-induced cellular damage

**Figure 2:**
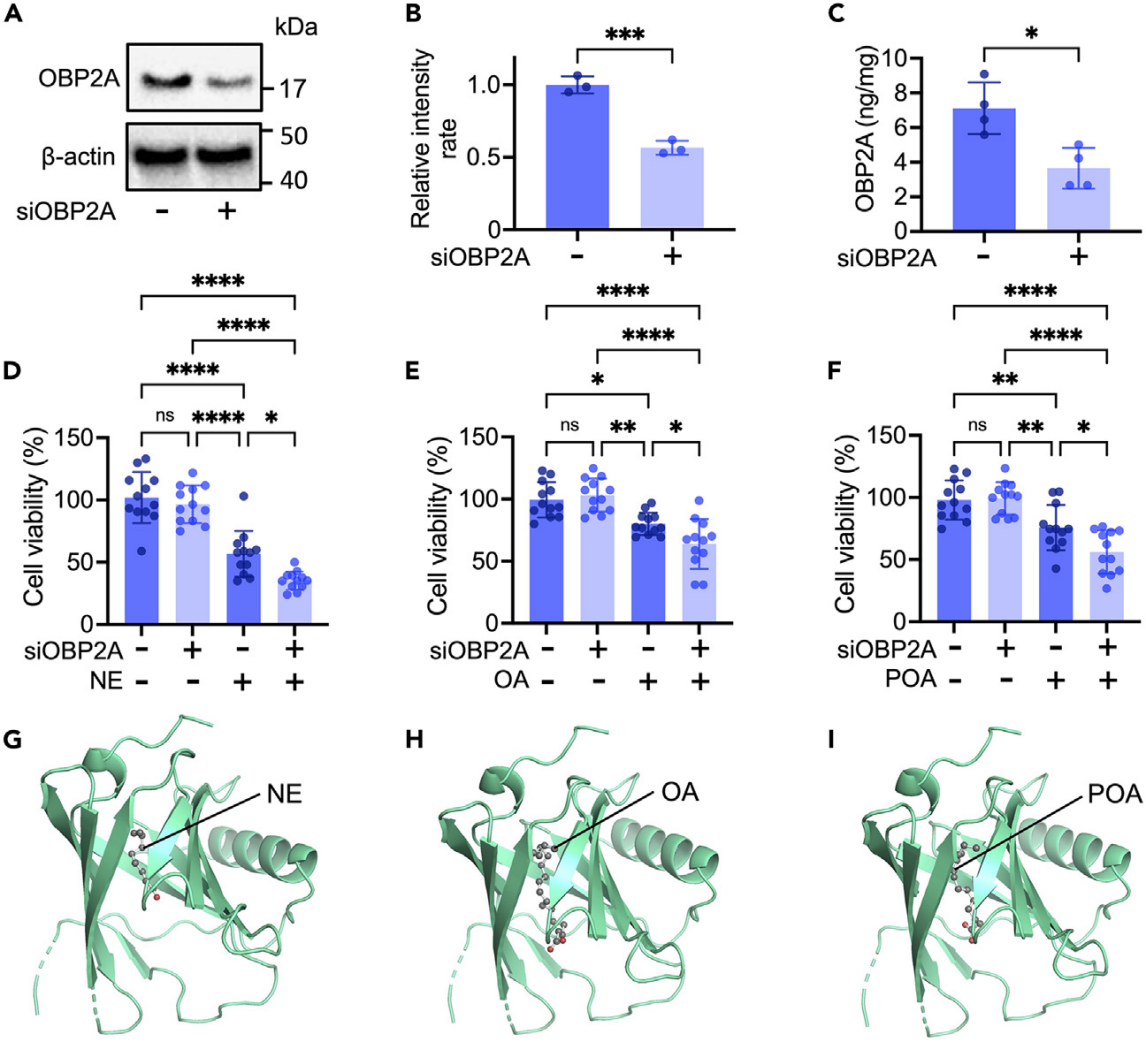
Effects of OBP2A on the reduction of cell viability induced by NE, OA, and POA (A) Western-blot analysis of OBP2A in cells. (B) Relative value of OBP2A western-blot signal (*n* = 3). (C) ELISA analysis of OBP2A in cells (*n* = 4). (D, E, and F) Viability of keratinocytes treated with NE, OA, or POA (*n* = 12). The cells were treated with scrambled siRNA or OBP2A siRNA. Anova F value = 47.41, *p* < 0.0001 for NE, Anova F value = 18.54, *p* < 0.0001 for OA, Anova F value = 19.04, *p* < 0.0001 for POA. (G, H, and I) Docking simulation of NE, OA, or POA to OBP2A. Gray: carbon atom, Red: oxygen atom, green: OBP2A (PDB ID: 4RUN). NE: *trans*-2-nonenal, OA: oleic acid, POA: palmitoleic acid. Bars and lines represent mean ± SD. ∗: *p* < 0.05, ∗∗: *p* < 0.01, ∗∗∗: *p* < 0.0005, ∗∗∗∗: *p* < 0.0001, ns: not significant in Student’s t test (B and C) and in ANOVA with Scheffé’s method (D, E, and F). See also Figure S2.

OBPs are capable of capturing many kinds of harmful aldehydes and fatty acids^15^^,^^19^^,^^23^^,^^24^ and serve as scavengers to protect mammalian olfactory cells.^13^^,^^16^ Harmful compounds include *trans*-2-nonenal (NE), which induces apoptosis of keratinocytes,^9^ and unsaturated fatty acids such as oleic acid (OA) and palmitoleic acid (POA), which impair murine skin barrier function.^25^^,^^26^ To test whether OBP2A is able to block the cytotoxic effects of aldehyde and unsaturated fatty acid on keratinocytes, we prepared keratinocytes in which OBP2A was markedly downregulated by means of small interfering RNA (siRNA) treatment, and significant a reduction of OBP2A expression was confirmed by western-blot analysis and ELISA (Figures 2A–2C). Application of either NE, OA, or POA significantly reduced the viability of intact keratinocytes, and their effects on OBP2A-knockdown keratinocytes were significantly increased (Figures 2D–2F). As shown in Figures 2G–2I, docking simulation indicated that NE, OA, and POA were captured in the barrel structure of OBP2A (binding affinity: −6.1 kcal/mol, −7.3 kcal/mol, and −6.2 kcal/mol, respectively). We also examined the effects of stearic acid (SA) and palmitic acid (PA), which are reported not to impair murine skin barrier function, and which differ from OA and POA only the absence of a double bond (the carbon chain length is the same).^25^ SA and PA did not affect the viability of intact or OBP2A-knockdown keratinocytes (Figures S2A and S2B) and docking simulation indicated that SA and PA were also captured in the barrel structure of OBP2A (binding affinity: −8.2 kcal/mol, and −7.2 kcal/mol, respectively) (Figures S2C and S2D).

Like OBPs, fatty acid binding proteins (FABPs) also belong to the lipocalin family. FABPs control intracellular transport and metabolism of fatty acids, and among them, FABP5 (also known as epidermal fatty acid binding protein, E-FABP) is predominantly expressed in epidermal keratinocytes.^27^ We also performed knockdown of FABP5. A significant reduction of FABP5 expression was confirmed (Figures S2E and S2F), but knockdown of FABP5 had no effect on NE, OA, or POA-induced cytotoxicity toward keratinocytes under our experimental conditions (Figures S2G–S2I). These findings suggest that OBP2A plays a key part in the protection of human keratinocytes against harmful aldehyde and fatty acids.

### OBP2A is involved in epidermal barrier formation

**Figure 3:**
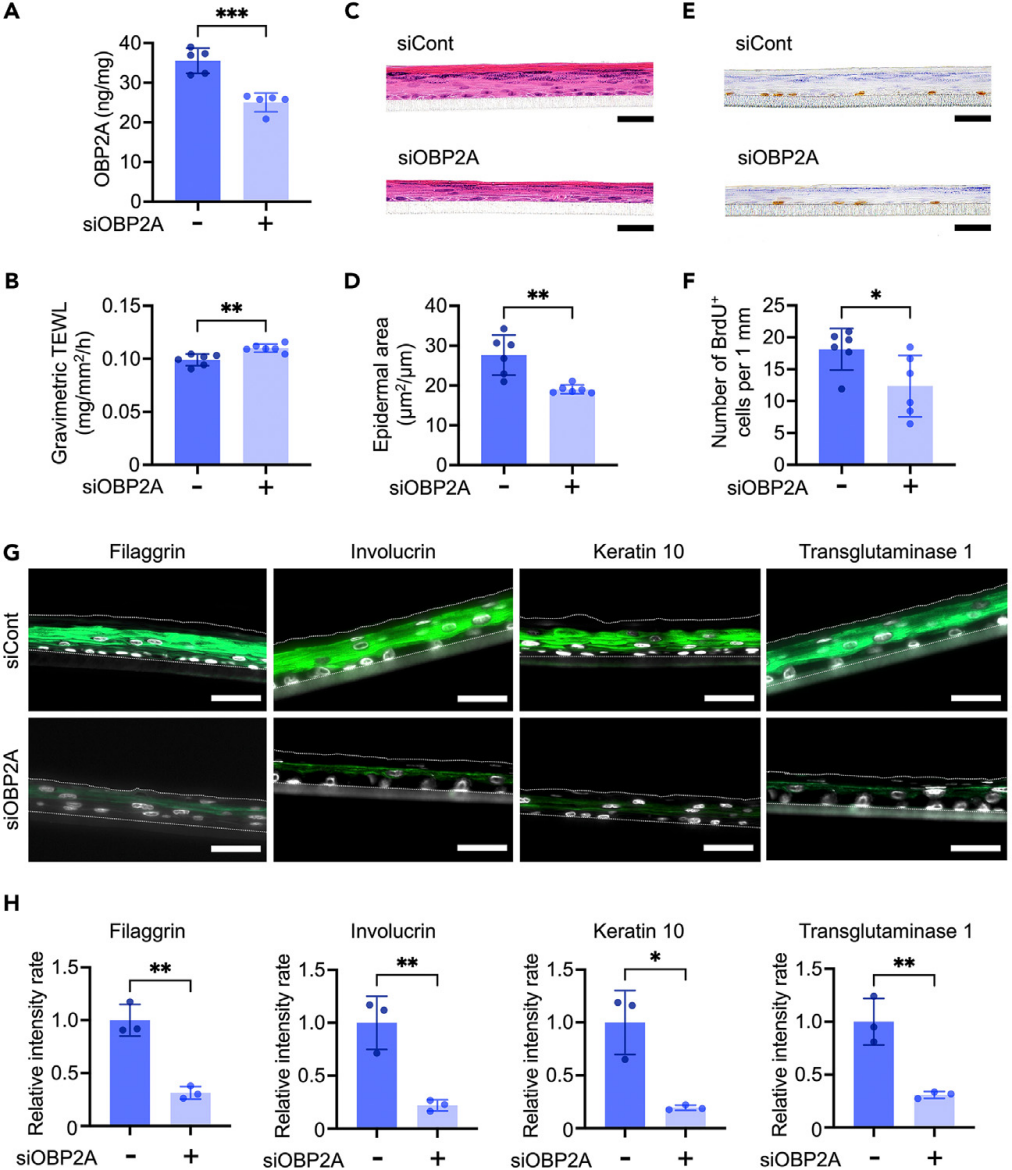
Dysfunction of epidermal differentiation in the OBP2A-knockdown 3DE-model (A) ELISA analysis of OBP2A in the 3DE-model (*n* = 5). (B) TEWL during 0-1h for the 3DE-model (*n* = 6). (C) Representative image of hematoxylin and eosin (H&E) stained 3DE-model. Bars = 40 μm. (D) Quantitation of epidermal area in the 3DE-model (*n* = 6). (E) Representative image of DAB-stained BrdU in the 3DE-model. Bars = 40 μm. (F) Quantitation of BrdU-positive cells in the 3DE-model (*n* = 6). (G) Immunofluorescence staining of epidermal barrier-related proteins (green) in the 3DE-model. Cell nuclei appear in white. Bars = 50 μm. (H) Relative fluorescence intensity of epidermal barrier-related proteins in the 3DE-model (*n* = 3). Bars and lines represent mean ± SD. ∗: *p* < 0.05, ∗∗: *p* < 0.01, ∗∗∗: *p* < 0.0005 in Student’s t test. See also Figure S3.

For further investigation of OBP2A functionality under steady-state conditions, we prepared a 3DE-model in which OBP2A was markedly downregulated by means of siRNA treatment (Figure 3A). The OBP2A-knockdown 3DE-model showed epidermal barrier dysfunction with increased transepidermal water loss (TEWL) (Figure 3B) and impairment of the epidermal phenotype, such as decreased epidermal thickness (Figures 3C and 3D). BrdU-positive proliferative cells were also significantly decreased in the OBP2A-knockdown 3DE-model (Figures 3E and 3F). In addition, several epidermal differentiation marker proteins, including filaggrin, involucrin, keratin 10, and transglutaminase 1, were significantly decreased (Figures 3G and 3H).^28^^,^^29^ Significant decreases of involucrin and keratin 10 were also confirmed in the OBP2A-knockdown 3DE-model by western-blot analysis (Figures S3A and S3B).

**Figure 4:**
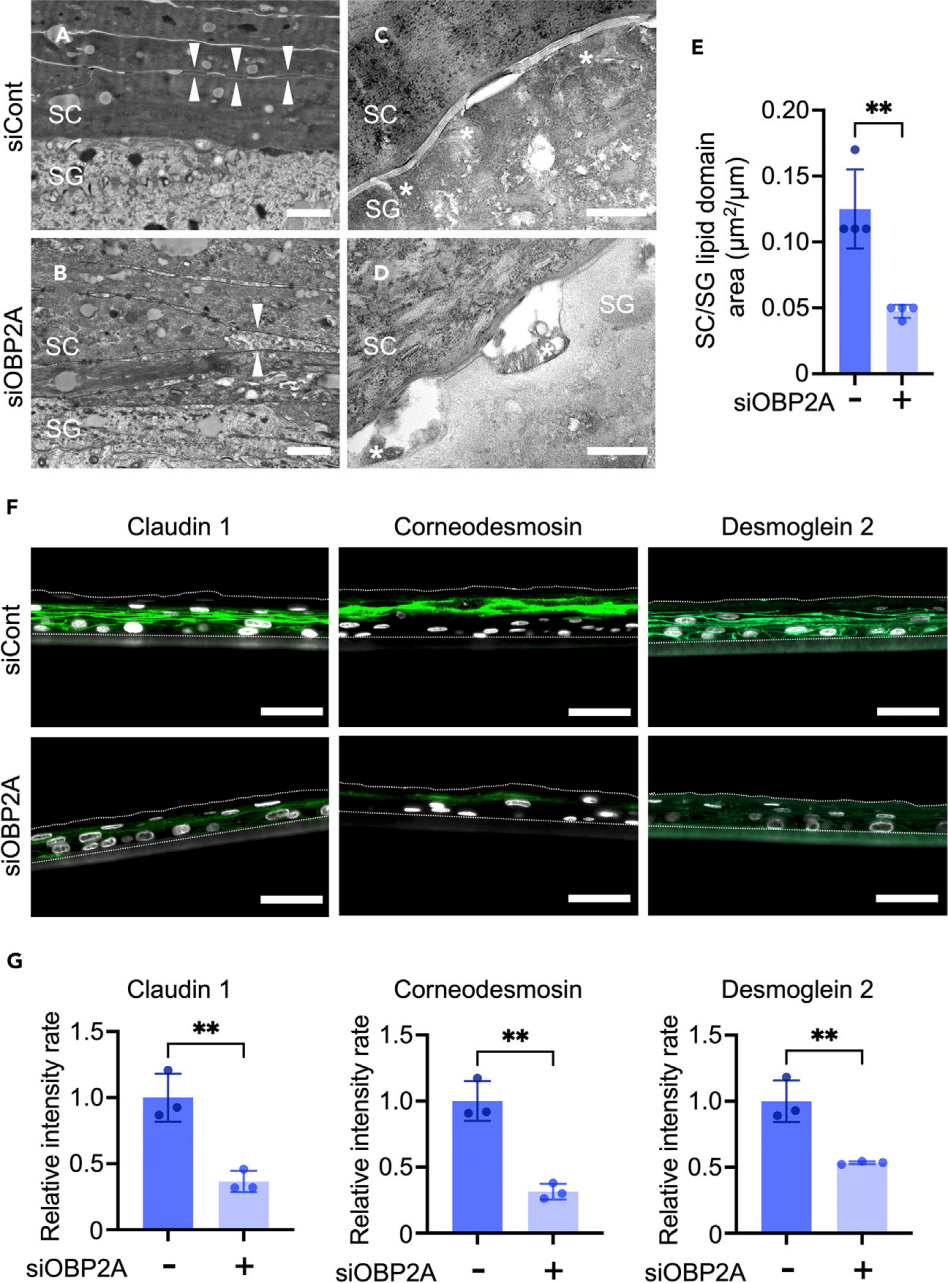
Dysfunction of desmosomes and lamellar secretion in the OBP2A-knockdown 3DE-model (A, B, C, and D) Electron-microscopic image of the 3DE-model treated with scrambled RNA (A and C) or OBP2A siRNA (B and D). (A and B): Osmium staining, Bars = 1 μm (C and D): Ruthenium staining, Bars = 400 nm. SG: stratum granulosum, SC: stratum corneum, white arrows: corneodesmosomes in the SC, ∗: intercellular lipid domains. (E) Quantitation of SG/SC lipid domains in the 3DE-model (*n* = 4). (F) Immunofluorescence staining of desmosomal proteins (green) in the 3DE-model. Cell nuclei appear in white. Bars = 50 μm. (G) Relative fluorescence intensity of desmosomal proteins in the 3DE-model (*n* = 3). Bars and lines represent mean ± SD. ∗∗: *p* < 0.01 in Student’s t test. See also Figure S3.

Next, we examined the alteration of skin barrier ultrastructure in the OBP2A-knockdown 3DE-model. Electron-microscopic observations revealed disruption of corneodesmosomal structure in the stratum corneum (Figures 4A and 4B). While lamellar granules fully filled with lipids were observed in the control 3DE-model, the lamellar granules in the OBP2A-knockdown 3DE-model contained vacant areas (Figures 4C and 4D). Furthermore, the lipid domain between the stratum granulosum and stratum corneum was significantly affected in the OBP2A-knockdown 3DE-model (Figure 4E). In accordance with the electron-microscopic observations, expression of desmosomal proteins, including claudin 1, corneodesmosin, and desmoglein 2, was significantly decreased in the OBP2A-knockdown 3DE-model (Figures 4F and 4G).^30^^,^^31^^,^^32^ Significant decreases of corneodesmosin and desmoglein 2 were also confirmed in the OBP2A-knockdown 3DE-model by western-blot analysis (Figures S3A and S3B). These findings suggest that OBP2A is essential for epidermal barrier formation.

### OBP2A is involved in epidermal cellular lipid metabolism

**Figure 5:**
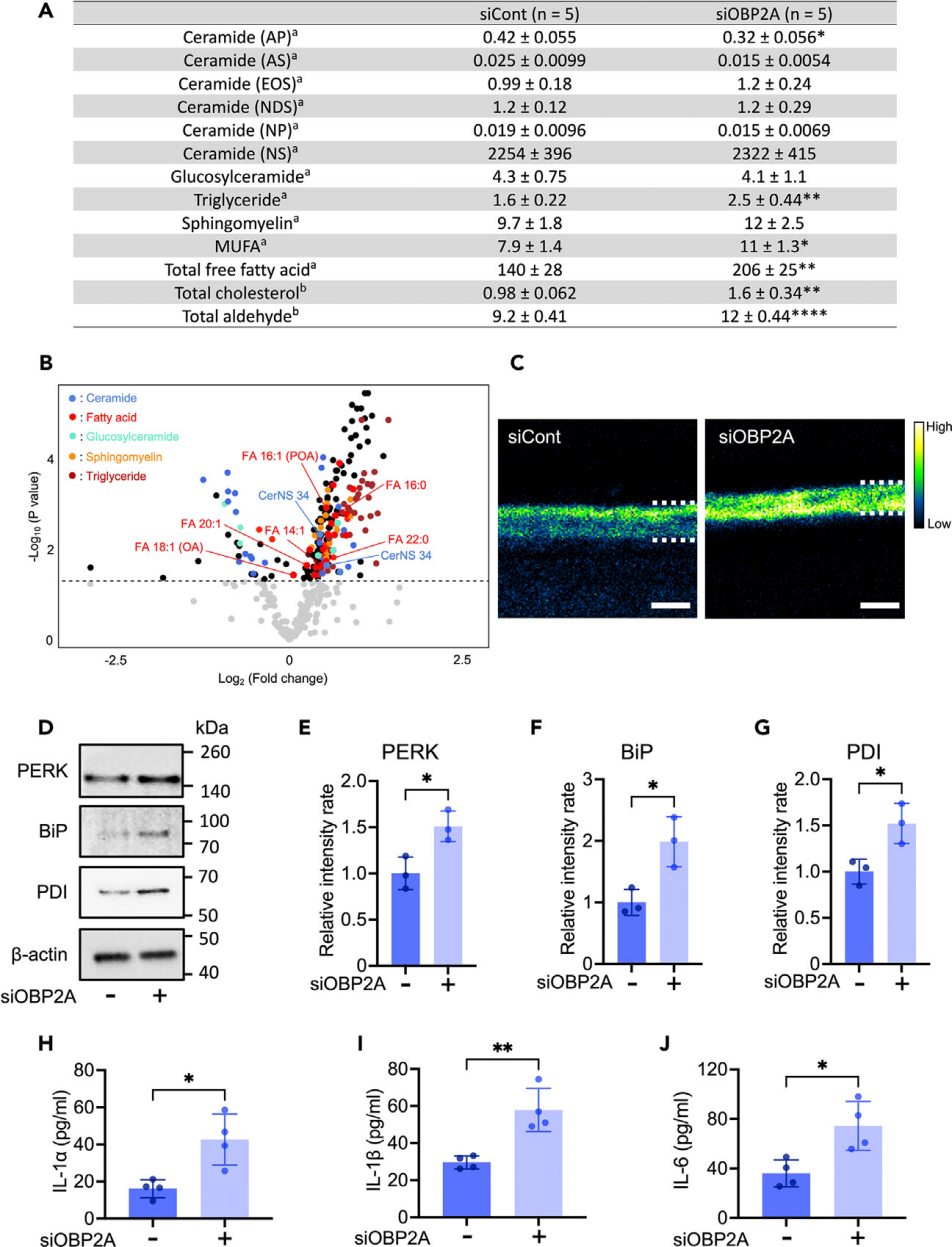
Influence of OBP2A-knockdown on composition of lipids, expression of ER stress marker proteins, and secretion of inflammatory cytokines in the 3DE-model (A) Quantitation of lipids detected by LC-MS/MS (*n* = 5). a: relative abundance to internal standard, b: nmol/mg. (B) Volcano plot of lipids detected by LC-MS/MS (*n* = 5). *p* < 0.05 was considered significant. (C) Image of total free fatty acid in the 3DE-model obtained with ToF-SIMS. Similar results were obtained in four independent experiments. Bars = 100 μm. (D) Western-blot analysis for the detection of PERK, BiP, PDI and β-actin in the 3DE-model. (E, F, and G) Relative value of the western-blot signal (*n* = 3). (H, I, and J) ELISA analysis of IL-1α, IL-1β, and IL-6 in cells (*n* = 4). Bars and lines represent mean ± SD. ∗: *p* < 0.05, ∗∗: *p* < 0.01, ∗∗∗∗: *p* < 0.0001 in Student’s t test. See also Figure S4, Tables S1, and S2.

OBP2A has high binding affinity for fatty acids.^15^^,^^19^^,^^23^^,^^24^ Therefore, to investigate whether cellular lipid metabolism disorders could cause the impairment of epidermal barrier formation in the OBP2A-knockdown 3DE-model, we performed lipidomics analysis using LC-MS/MS. We found that 192 lipids were significantly increased and 33 lipids were significantly decreased in the OBP2A-knockdown 3DE-model (Table S1). Not only the amount of monounsaturated fatty acids (MUFA), including OA and POA, but also the total amount of free fatty acid (FA) was significantly increased in the OBP2A-knockdown 3DE-model (Figures 5A and 5B). The total amount of cholesterol, aldehyde, and triglyceride was also significantly increased (Figure 5A), while the total amount of ceramide AP was significantly decreased (Figure 5A). Although there was no significant difference in the total amounts of glucosylceramide and sphingomyelin in the OBP2A-knockdown 3DE-model (Figure 5A), the amounts of several glucosylceramides were significantly altered and the amounts of several sphingomyelins were significantly increased in the OBP2A-knockdown 3DE-model (Figure 5B; Table S1). Significant increases in free fatty acids (FA 14:1, FA 16:0, FA 16:1, FA 18:1, FA 20:1, FA 22:0) and short C34 ceramide NS, which have been reported to be increased in atopic dermatitis skin,^33^^,^^34^ were observed in the OBP2A-knockdown 3DE-model (Figure 5B). It should be noted that previous studies have reported differences of ceramide profile in the stratum corneum,^34^^,^^35^^,^^36^ but our analysis covers the ceramide profile in the whole epidermis.

Next, we visualized the spatial distribution of free fatty acids in the OBP2A-knockdown 3DE-model, using time-of-flight secondary ion mass spectrometry (ToF-SIMS) imaging. Consistent with previous reports,^37^^,^^38^ free fatty acids were mainly observed in the epidermal upper layer of the normal 3DE-model (Figures 5C and S4). However, free fatty acids were irregularly distributed throughout the whole epidermal area in the OBP2A-knockdown 3DE-model (Figures 5C and S4). The endoplasmic reticulum (ER) is a major hub for cellular lipid metabolism, and persistent free fatty acid accumulation leads to ER stress.^39^^,^^40^ Therefore, we investigated the relationship between impairment of lipid metabolism and ER stress in the OBP2A-knockdown 3DE-model. The expression levels of PERK, Bip, and PDI, which are markers of ER stress,^41^ were significantly increased in the OBP2A-knockdown 3DE-model (Figures 5D–5G). Because ER stress leads to sterile inflammation, which is commonly found in various disorders,^42^^,^^43^ we measured the levels of inflammatory cytokines in the OBP2A-knockdown 3DE-model. Significant increases of IL-1α, IL-1β, and IL-6 were observed (Figures 5H–5J). These findings suggest that ER stress is caused, at least in part, by the impairment of cellular lipid metabolism in human keratinocytes when OBP2A is inhibited.

### OBP2A is relevant to atopic dermatitis

**Figure 6:**
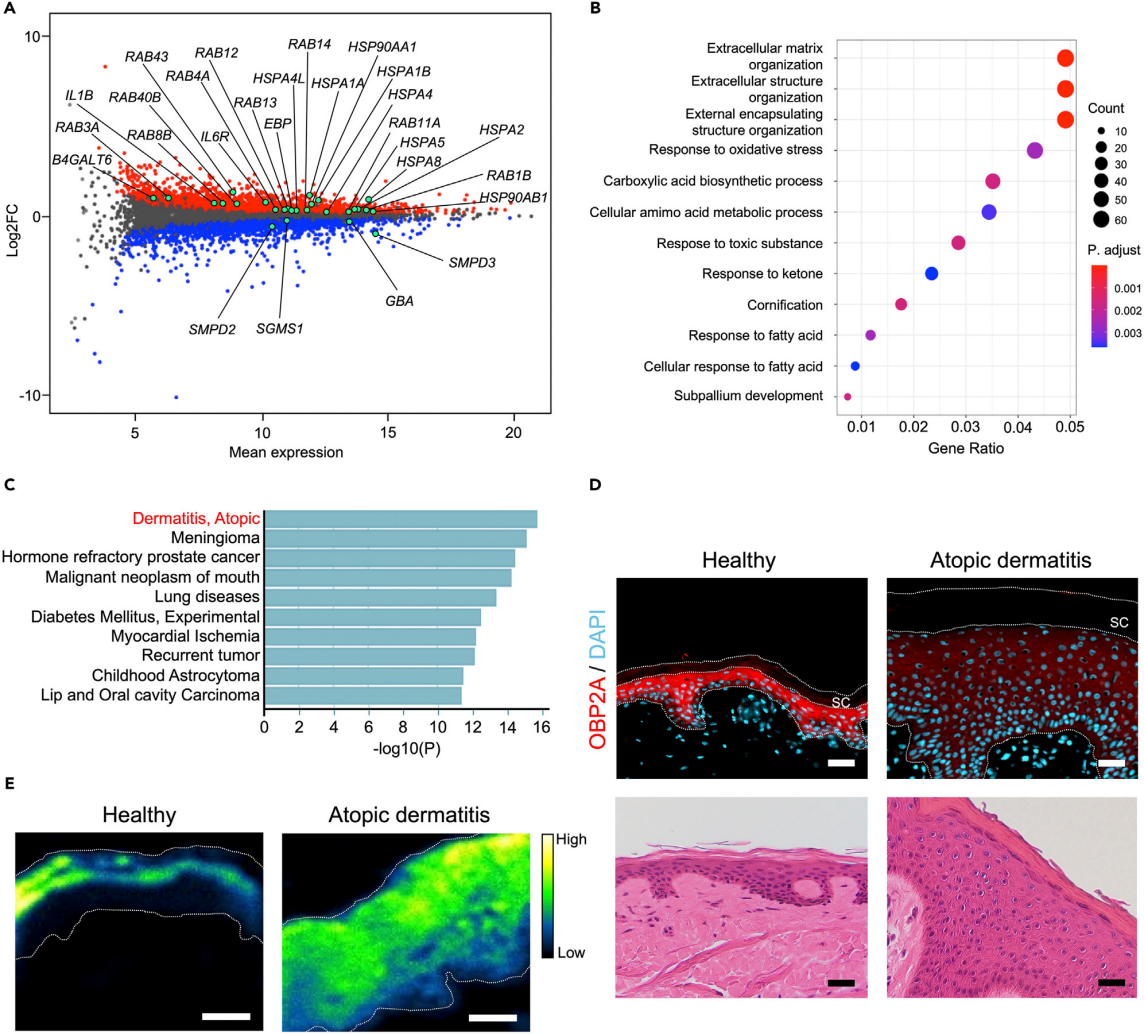
Dysfunction of epidermal differentiation induced by OBP2A-knockdown associated with atopic dermatitis (A) MA plot of differential gene expression between scrambled siRNA and OBP2A siRNA-treated 3DE-model. Significantly differentially expressed genes (DEG) (FDR <0.01) are indicated by red or blue dots (*n* = 5). Genes of interest are indicated by green dots. (B) Biological process terms gene ontology (GO:BP) analysis for DEG with more than 1.5-fold change. (C) Human gene-disease associations terms gene ontology analysis for DEG with more than 1.5-fold change. (D) H&E staining and immunofluorescence staining of OBP2A in healthy or atopic dermatitis lesional skin. Bars = 30 μm. (E) Image of total free fatty acid in healthy or atopic dermatitis lesional skin obtained with ToF-SIMS. Similar results were obtained in four independent experiments. Bars = 100 μm. See also Figures S5, S6, and Table S2.

**Figure 7:**
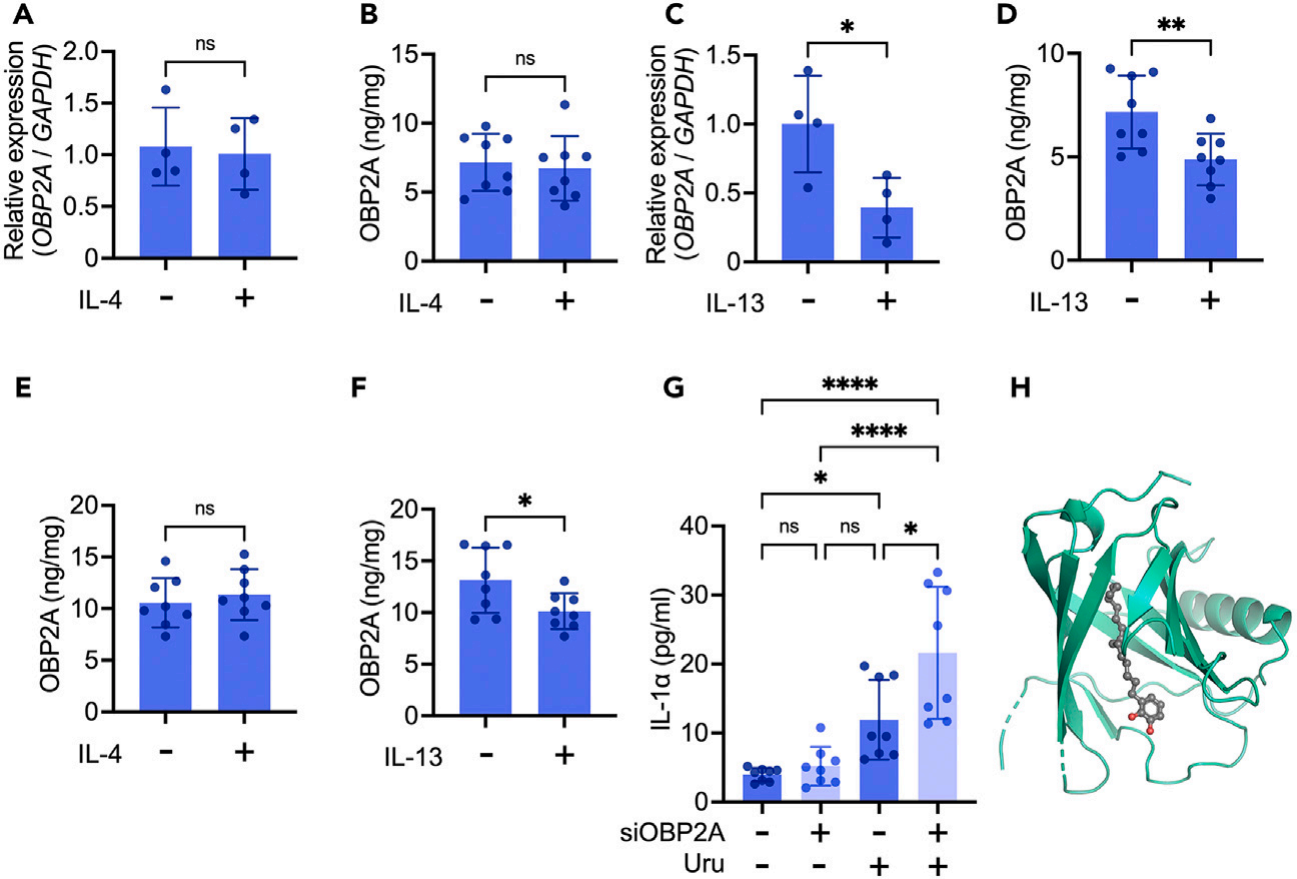
Effect of IL-4, IL-13, and urushiol on OBP2A expression and IL-1α secretion in keratinocytes (A and C) qPCR analysis of OBP2A in cells treated with IL-4 or IL-13 (*n* = 4). (B and D) ELISA analysis of OBP2A in cells treated with IL-4 or IL-13 (*n* = 8). (E and F) ELISA analysis of OBP2A in the 3DE-model treated with IL-4 or IL-13 (*n* = 8). (G) ELISA analysis of IL-1α in cells treated with urushiol (Uru) (*n* = 8). Anova F value = 15.63, *p* < 0.0001. (H) Docking simulation of urushiol to OBP2A. Gray: carbon atom, red: oxygen atom, green: OBP2A (PDB ID: 4RUN). Bars and lines represent mean ± SD. ∗: *p* < 0.05, ∗∗: *p* < 0.01, ∗∗∗: *p* < 0.0005, ns: not significant in Student’s t test (A, B, C, D, E, and F) and in ANOVA with Scheffé’s method (G).

To further clarify the role of OBP2A in human keratinocytes, we performed RNA sequencing (RNA-seq) of OBP2A-knockdown 3DE-model samples. We found that 2629 genes were significantly upregulated and 2540 genes were significantly downregulated (Figure 6A). Among them, we found that many kinds of ER stress and chaperone molecule-related genes, including *HSP90AA1*, *HSP90AB1*, *HSPA1A*, *HSPA1B*, *HSPA2*, *HSPA4*, *HSPA4L*, *HSPA5*, and *HSPA8*,^44^^,^^45^^,^^46^ intracellular membrane trafficking molecule-related RAB family genes, including *RAB1B*, *RAB3A*, *RAB4A*, *RAB8B*, *RAB11A*, *RAB12*, *RAB13*, *RAB14*, *RAB40B*, *RAB43*, and inflammatory cytokine-related genes, including *IL1B* and *IL6R*, were significantly upregulated in the OBP2A-knockdown 3DE-model (Figure 6A), indicating a disturbance of epidermal protein folding and transport. In accordance with the results of lipidomics analysis, expression of glucosylceramide metabolic genes, *EBP (GLB1)*, *B4GALT6*, and *GBA,* was significantly altered in the OBP2A-knockdown 3DE-model. *SGMS1*, *SMPD2*, and *SMPD3,* which mediate ceramide synthesis from sphingomyelin were also significantly downregulated in the OBP2A-knockdown 3DE-model (Figure 6A). Gene ontology (GO) term enrichment analysis also showed that extracellular organization, cornification, and response to fatty acid-related genes were altered in the OBP2A-knockdown 3DE-model (Figure 6B). Interestingly, these genes are also related to atopic dermatitis (Figure 6C). Therefore, we next examined OBP2A expression in atopic dermatitis lesional skin samples, and found that it was markedly decreased compared with that in healthy skin (Figures 6D and S5A). We also confirmed a significant reduction of OBP2A expression in publicly available DNA microarray data of atopic dermatitis skin compared with healthy skin (GSE16161)^47^ (Figure S5B). Consistent with our data obtained in the OBP2A-knockdown 3DE-model, ToF-SIMS imaging data also showed a distribution of free fatty acid in the upper layer of healthy skin, whereas irregularly distribution of free fatty acid was observed in atopic dermatitis skin (Figures 6E and S5C). Likewise, disruption of corneodesmosomal structure and lamellar bodies was also observed in atopic dermatitis skin by electron microscopy (Figure S6).^48^^,^^49^ To get insight into the mechanism of the decline in OBP2A expression in atopic dermatitis lesional skin, we measured changes in OBP2A expression in human keratinocytes treated with type 2 cytokine IL-4 or IL-13, known to be involved in the pathogenesis of atopic dermatitis.^50^ Neither the transcription nor the protein level of OBP2A expression was significantly changed upon IL-4 treatment (Figures 7A and 7B), but IL-13 treatment significantly suppressed both the transcription and protein level of OBP2A in human keratinocytes (Figures 7C and 7D). IL-13 treatment also significantly suppressed the protein level of OBP2A in the 3DE-model, while IL-4 treatment did not (Figures 7E and 7F). Patients with atopic dermatitis are known to be more vulnerable to allergen sensitization in skin.^12^ Since it has been reported that urushiol, a well-characterized skin allergen, induces secretion of the inflammatory cytokine IL-1α, which is known to be abundant in keratinocytes,^51^^,^^52^^,^^53^ we examined whether the action of urushiol is affected by OBP2A-knockdown. As expected, the level of IL-1α was significantly enhanced in OBP2A-knockdown keratinocytes upon treatment with urushiol (Figure 7G). Docking simulation suggested that urushiol might be captured by OBP2A (binding affinity: −7.6 kcal/mol) (Figure 7H).

## Discussion

The expression patterns of OBPs are organ-specific,^17^^,^^18^ suggesting that different OBPs may have different roles. Our present results show that OBP2A is highly expressed in human epidermal keratinocytes, and suggest a previously uncharacterized role of OBP2A in skin homeostasis. We found that cellular resistance to cytotoxic aldehyde and lipids, which are speculated to be captured by OBP2A, is reduced in keratinocytes when OBP2A is inhibited. This is consistent with the fact that OBP2A has a high binding affinity for aldehydes and fatty acids,^15^^,^^19^^,^^23^^,^^24^ and also with previous reports that porcine and bovine OBPs protect cells from damage caused by aldehyde and lipids.^54^^,^^55^ LCN1, human tear lipocalin, is also thought to be a scavenger of harmful lipophilic substances and might act as a general protection factor in tears.^56^ Lipocalin also plays a role in the response to environmental stresses in plants.^57^ Thus, OBPs are capable of protecting against a wide range of harmful small hydrophobic molecules in the environment.^15^^,^^23^^,^^58^ OBP2A expression in the upper layer of the skin might be reasonable to capture various harmful small hydrophobic molecules in the environment. Although SA and PA did not affect the cell viability regardless of OBP2A-knockdown, docking simulation showed that OBP2A might bind with SA and PA. It has been reported that OBPs are not only scavengers of harmful small hydrophobic molecules, but also transporters of odorants and pheromones to olfactory cells in the mucus.^13^^,^^16^ Further study is required to clarify the precise mechanisms of OBP2A action after ligand binding.

OBPs are also involved in tissue development processes in insects.^19^^,^^20^^,^^21^^,^^22^ It has also been reported that lipocalins and their ligands are transferred into the cell by endocytosis and regulate cell differentiation and proliferation.^59^ In this study, we found that the OBP2A-knockdown 3DE-model showed dysfunction of epidermal barrier formation. Lipidomics analysis revealed that the amounts of major compounds contributing to epidermal barrier construction, such as cholesterols, ceramides, and free fatty acids, were significantly altered and the free fatty acid distribution pattern in the epidermis was disrupted by OBP2A-knockdown. This is consistent with the observation that accumulation of OA, POA, and aldehyde in the 3DE-model caused by silencing of OBP2A is associated with dysfunction of epidermal barrier formation. In the OBP2A-knockdown 3DE-model, whereas total MUFA, cholesterol, triglyceride, several free fatty acids (FA 14:1, FA 16:0, FA 16:1, FA 18:1, FA 20:1, FA 22:0), and short C34 ceramide NS were increased, ceramide AP was decreased. Notably, similar compound changes occur in atopic dermatitis skin^33^^,^^34^^,^^35^^,^^36^^,^^60^^,^^61^^,^^62^^,^^63^^,^^64^^,^^65^ and lead to an increase of TEWL.^36^ We also confirmed the induction of ER stress and secretion of inflammatory cytokines in the OBP2A-knockdown 3DE-model, and these phenomena are deeply related to dysfunction of desmosomal structure,^33^^,^^44^^,^^45^ lamellar body secretion,^66^ and keratinocyte differentiation,^33^ which are also observed in atopic dermatitis skin.^67^^,^^68^^,^^69^^,^^70^^,^^71^^,^^72^ Indeed, we found that ER stress and chaperone molecule-related genes and inflammatory cytokine genes were upregulated in the OBP2A-knockdown 3DE-model. The RAB family is required for normal protein transport and also plays crucial roles in the polarization of claudin 1, corneodesmosin, desmoglein 2, and filaggrin.^73^^,^^74^^,^^75^^,^^76^ Since RAB family genes were upregulated in the OBP2A-knockdown 3DE-model, abnormal protein transport may also contribute to the dysfunction of epidermal barrier formation in this model. Since fatty acid accumulation leads to ER stress,^39^^,^^40^ and ER stress induces an inflammation response,^42^^,^^43^ fatty acid accumulation induced by OBP2A deficiency might be one of the causes of epidermal homeostasis dysfunction.

Consistent with the results of lipidomics and transcriptome analysis in OBP2A-knockdown 3DE-model, we observed a reduction of OBP2A expression and an abnormal free fatty acid distribution in atopic dermatitis lesional skin. It has been reported that OBP expression is reduced in canine atopic dermatitis.^77^ Furthermore, secretion of IL-4 and IL-13, one of the major inflammatory cytokines of atopic dermatitis, from Th2 cells is induced at least in part by IL-1α, IL-1β, and IL-6.^50^ Interestingly, we found that only IL-13 but not IL-4 reduced the OBP2A expression in keratinocytes. It has been reported that IL-13 binds to IL-13Rα2 and activates AP-1 in addition to activation of JAK/STAT pathways.^78^ AP-1 is highly expressed in keratinocytes of atopic dermatitis,^79^^,^^80^ and application of T-5224, c-Fos/AP-1 inhibitor ameliorates clinical manifestations of atopic dermatitis-like dermatitis in mice.^81^ These results provide a possibility that OBP2A expression might be under AP-1 regulation. However, JAK/STAT pathways are still not fully clarified and we cannot exclude the possibility of JAK/STAT pathway involvement. Future studies are necessary to understand regulation mechanism of OBP2A expression.

Our data also suggested that OBP2A is involved in the skin’s sensitivity to urushiol. Considering this result, together with the fact that OBP2A binds with eugenol, citronellol, and geraniol, which are known as odorant allergens,^15^ OBP2A may play a role in protecting the skin from allergens. Lipocalin is thought to be associated with innate immunity and allergy in humans,^82^ and is known to regulate immune response and to be associated with inflammatory diseases in mammalians and insects.^21^^,^^83^^,^^84^ Moreover, lipocalins including OBP can inactivate viral DNA, as well as showing antibacterial, and antifungal activity.^56^^,^^83^

In conclusion, our findings reveal a previously uncharacterized role of OBP2A in skin homeostasis. OBP2A may influence epidermal barrier formation via its involvement in epidermal lipid metabolism and blocking the effects of harmful small hydrophobic molecules present in the environment.

### Limitations of the study

First, we focused only on OBP2A among OBP family members in this study. However, OBP2B has high similarity with OBP2A (90% amino acid sequence similarity). Although, functional difference of OBP2A and OBP2B is speculated in previous studies, the involvement of OBP2B should also be investigated. Second, the effects on other harmful small hydrophobic molecules or allergens should be tested to better explore this study. Third, the mechanism through which OBP2A regulates lipid metabolism in keratinocytes remains to be established in detail. Finally, atopic dermatitis is influenced by many factors and mechanisms, so it will be necessary to investigate a larger number of samples of atopic dermatitis lesional skin to confirm the role of OBP2A in atopic dermatitis.

## Resource availability

### Lead contact

Further information and requests for resources and reagents should be directed to and will be fulfilled by the lead contact, S.N. (shinobu.nakanishi@shiseido.com).

### Materials availability

This study did not generate new unique reagents and all materials in this study are commercially available.

### Data and code availability

• Raw RNA-seq data have been deposited in NCBI Sequence Read Archive (SRA) are publicly available. Data identification number is listed in the key resources table.
• This article does not report original code.
• Any additional information required to reanalyze the data reported in this paper is available from the lead contact upon request.

## Acknowledgments

We thank Ms. Makiko Ogura for technical support. S.N., T.H., K.M., and A.M. were supported by Shiseido Co. Ltd. Graphic abstract was created with BioRender.

## Author contributions

S.N. mainly designed the research study, performed the experiments and wrote the manuscript: T.H. discussed the results and analyzed the data and wrote the manuscript: K.M. performed LC-MS/MS analysis: A.M. and M.D. discussed the results. All authors edited the manuscript.

## Declaration of interests

The authors declare no competing interests.

## STAR★Methods

### Key resources table

**Table:** 

REAGENT or RESOURCE	SOURCE	IDENTIFIER
**Antibodies**		

β-actin Antibody	Santa Cruz Biotechnology	sc-47778
Bip (C50B12) Rabbit mAb	Cell Signaling Technology	3177
Claudin 1 Polyclonal Antibody	Invitrogen	71–7800
CDSN antibody	Proteintech	13184
Anti-Corneodesmosin	abcam	ab204235
Human Desmoglein-2 Antibody	R&D Systems	MAB947
FABP5 (D1A7T) Rabbit mAb	Cell Signaling Technology	39926
Anti Filaggrin (AKH1)	Santa Cruz Biotechnology	sc-66192
Anti-Involucrin	abcam	ab53112
Anti-Keratin 10	Fitzgerald Industries International	20R-CP002
Anti-Cytokeratin 10 antibody [EP1607IHCY]	abcam	ab76318
OBP2A antibody	Biorbyt	orb582625
OBP2A antibody	Biorbyt	orb37374
PDI (C81H6) Rabbit mAb	Cell Signaling Technology	3501
PERK (D11A8) Rabbit mAb	Cell Signaling Technology	5683
Anti TGase1 (E−6)	Santa Cruz Biotechnology	sc-166467
Anti-BrdU [BU1/75 (ICR1)]	Abcam	ab6326
Polyclonal biotinylated rabbit anti-rat Ig	DAKO	#E0468
Alexa Fluor 488 AffiniPure Donkey Anti-Guinea Pig IgG (H + L)	Jackson ImmunoResearch	AB_2340472
Donkey anti-Rabbit IgG (H + L) Highly Cross-Adsorbed Secondary Antibody, Alexa Fluor 488	Invitrogen	A21206
Donkey anti-Rabbit IgG (H + L) Highly Cross-Adsorbed Secondary Antibody, Alexa Fluor 594	Invitrogen	A21207
iFluor 594 PSA Imaging Kit with goat anti-rabbit IgG	AAT Bioquest	#45230

**Biological samples**		

Fresh Human Full Thickness Skin Disc	Biopredic International	DISC1D8
Atopic dermatitis skin fresh frozen	BioIVT	147803, 223571, HUMANSPBIO-0019461, HUMANSPBIO-0019461

**Chemicals, peptides, and recombinant proteins**		

Recombinant Human IL-4 Protein	R&D Systems	6507-IL/CF
Recombinant Human IL-13 Protein	R&D Systems	213-ILB/CF
PENTADECYLCATECHOL, 3-	FUJIFILM Wako Pure Chemical Corporation	492-89-7, ASB-00021675-005
*trans*-2-Nonenal	Toronto Research Chemicals	18829-56-6, N649710
Oleic Acid	Nakarai Tesque	112-80-1, 25630-51
Palmitoleic Acid	FUJIFILM Wako Pure Chemical Corporation	373-49-9, 10009871
Stearic Acid	Tokyo Chemical Industry	57-11-4, S0163
Palmitic Acid	Tokyo Chemical Industry	57-10-3, P1145
EquiSPLASH	Avanti Polar Lipids	330731-1EA

**Critical commercial assays**		

RNeasy Mini Kit	QIAGEN	74104
SuperScript IV VILO Master Mix	Invitrogen	11756050
LightCycler 480 Probes Master	Roche Diagnostics	04707494001
Polyolefin micro sealing tape	3 M Japan Limited	9793
2.5g/L-Trypsin/1mmol/L-EDTA Solution	Nacalai tesque	35554–64
Trypsin Neut Solution	Kurabo Industries	HK-3220
Trypan blue solution	Nacalai tesque	20577–34
Lipofectamine RNAiMAX Transfection Reagent	Thermo Fisher Scientific	13778150
Opti-MEM I Reduced Serum Medium	Thermo Fisher Scientific	31985062
CnT-Prime Epithelial Proliferation Medium	CELLnTEC	CnT-PR
CnT-Prime Epithelial 3D Airlift Medium	CELLnTEC	CnT-PR-3D
Dulbeccos Phosphate Buffered Saline, With MgCl2 and CaCl2, liquid, sterile-filtered, suitable for cell culture	Sigma-Aldrich	D8662
CELLstart Substrate	Thermo Fisher Scientific	A10142-01
Millicell Hanging Cell Culture Insert, PET 0.4 μm, 24-well, 48/pk	Millipore	PTHT24H48
RIPA Buffer	Nacalai tesque	16488–34
Protease Inhibitor Cocktail (100×)	Nacalai tesque	04080–24
Bio-Rad Protein Assay Standard I	BIO-RAD	#500-0005
DC Protein Assay Reagent A	BIO-RAD	#5000113
Protein Assay Reagent B	BIO-RAD	#5000114
DC Protein Assay Reagent S	BIO-RAD	#500-0115
Human Odorant Binding Protein 2A (OBP2A) ELISA Kit	MyBioSource	#MBS2024980
Human Odorant Binding Protein 2B (OBP2B) ELISA kit	MyBioSource	#MBS9322367
Human IL-1 alpha/IL-1F1 Quantikine ELISA Kit	R&D Systems	DLA50
Human IL-1 beta/IL-1F2 Quantikine ELISA Kit	R&D Systems	DLB50
Human IL-6 Quantikine ELISA Kit	R&D Systems	D6050
iBlot 2 Transfer Stacks, PVDF, regular size	Invitrogen	IB24001
iBind Cards	Invitrogen	SLF1010
NuPAGE Bis-Tris Mini Protein Gels, 4–12%, 1.0–1.5 mm	Invitrogen	NP0323BOX
iBind Solution Kit	Invitrogen	SLF1020
AP Chemiluminescent Substrate	Invitrogen	WP20002
Spectra Multicolor Broad Range Protein Ladder	Thermo Fisher Scientific	26634
VECTASTAIN ABC Rabbit IgG Kit	Vector Laboratories	PK-4001
DAB Substrate	Roche Diagnostics	11718096001
Aldehyde Assay Kit II (Colorimetric-Blue)	abcam	Ab219923
Amplite Cholesterol Quantitation Kit Red Fluorescence	AAT Bioquest	#40006
Glass Slides for MALDI Imaging	Bruker Daltonics	8237001
Tissue-Tek O.C.T. Compound	Sakura Finetek Japan	4583
ProLong Gold antifade reagent	Invitrogen	P36930
Amicon Ultra Centrifugal Filter, 3 kDa MWCO	Millipore	UFC5003
Novex AP Rabbit Chemiluminescent Detection Kit	Invitrogen	SLF1022
Novex AP Mouse Chemiluminescent Detection Kit	Invitrogen	SLF1021
DAPI	Invitrogen	#D3571

**Deposited data**		

GEO database	National Center for Biotechnology Information	https://www.ncbi.nlm.nih.gov/geo/query/acc.cgi?acc=GSE16161
RNA-seq data	This paper	PRJNA1135367
Human reference sequence	illumina	https://support.illumina.com/sequencing/sequencing_software/igenome.html
GTF annotation file	illumina	https://support.illumina.com/sequencing/sequencing_software/igenome.html
Protein databank	RCSB PDB	https://www.rcsb.org/
PubChem	National Center for Biotechnology Information	https://pubchem.ncbi.nlm.nih.gov/

**Experimental models: Cell lines**		

Normal human epithelial keratinocytes	Kurabo	KK-4009

**Oligonucleotides**		

Primer	Invitrogen	Table S3
ON-TARGETplus Human OBP2A siRNA	GE Healthcare Dharmacon	J-010028-17, J-010028-18, J-010028-19, J-010028-20
ON-TARGETplus Human FABP5 siRNA	GE Healthcare Dharmacon	J-008710-07, J-008710-08, J-008710-09, J-008710-10
ON-TARGETplus Non-targeting Pool	GE Healthcare Dharmacon	D-001810-10-05

**Software and algorithms**		

Zen Blue	Zeiss	https://www.zeiss.com/microscopy/en/products/software/zeiss-zen.html
GraphPad Prism	GraphPad	https://www.graphpad.com/scientific-software/prism/
Biorender	Biorender	https://biorender.com/
ImageJ	National Institutes of Health	https://imagej.net/ij/
R	The R foundation	https://www.r-project.org/
Python	Python Software Foundation	https://www.python.org/
FastQC	Babraham Bioinformatics	https://www.bioinformatics.babraham.ac.uk/projects/fastqc/
iDEP.96	South Dakota State University	http://bioinformatics.sdstate.edu/idep96/
Metascape	Zhou Y et al.	https://metascape.org/gp/index.html#/main/step1
Open Babel	Open Babel development team	http://openbabel.org/
PyMOL	Schrödinger	https://www.pymol.org/
AutoDock Tools	Center for Computational Structural Biology	https://autodocksuite.scripps.edu/
MS-DIAL	Github	http://prime.psc.riken.jp/compms/msdial/main.html, version 4.48
Reifycs file converter	Reifycs Inc.	https://www.reifycs.com/abfconverter/
MetaboAnalyst		https://www.metaboanalyst.ca/

### Experimental model and study participant details

#### Cells and cell culture

Normal human epithelial keratinocytes were purchased from Kurabo (Osaka, Japan) and cultured in EPILIFE-KG2 (Kurabo, Osaka, Japan). Keratinocytes were first cultured to 100% confluency in low-Ca^2+^ (0.06 mM) medium with or without siRNA for 24h, then differentiated in high-Ca^2+^ (1.8 mM) medium, and used for experiments. Carrier-free IL-4 or IL-13 (25 ng/mL) (R&D Systems, Minnesota, USA), or urushiol (3-pentadecylcatechol) (10 μM) (FUJIFILM Wako Pure Chemical Corporation, Tokyo, Japan) was applied.

#### Human skin tissue

Healthy and atopic dermatitis human skin tissues were purchased from Biopredic International (Rennes, France) via KAC Co., Ltd. (Kyoto, Japan) and BioIVT (NY, USA) via Cosmo Bio Co., Ltd. (Tokyo, Japan), respectively. The samples had been obtained following plastic surgery, with informed consent. The healthy excised skin was dermatomed to 340–440 μm thickness (containing epidermis and dermis), and then discs (10 mm in diameter, thickness about 2 mm) were punched out, transferred to our laboratory, and immediately frozen in liquid nitrogen. The atopic dermatitis skin samples were obtained, with informed consent, as punch biopsies (2 mm in diameter, thickness about 2 mm), which immediately frozen in liquid nitrogen. Then, the frozen samples were transferred to our laboratory. Four samples of healthy skin tissues from abdomen (42, 45, 50, and 53 years old, Caucasian females) and atopic dermatitis skin tissues from buttock, elbow or back (34, 39, 61, and 63 years old, Caucasian females) were used for the study. This study was approved by the ethics committee of Shiseido in accordance with the guidelines of the National Institute of Health.

### Method details

#### Quantitative real-time PCR (qPCR)

Total RNA from human keratinocytes was isolated using RNeasy mini kits (QIAGEN, Hilden, Germany), and complementary DNA (cDNA) synthesis from 1 μg of total RNA was performed using SuperScript VILO Master Mix (Invitrogen, CA, USA). The PCR reactions were performed on a LightCycler 480 System II (Roche Diagnostics Inc, Basel, Switzerland) using LightCycler 480 Probes Master (Roche Diagnostics Inc, Basal, Switzerland), cDNA and specific primer pairs: *GAPDH*: forward, gaaggtgaaggtcggagtc and reverse, gaagattggtgatgggatttc; *OBP2A*: forward, gagcctggcaaattcagc and reverse, tctttgcagtaaaagacgtagtcg; *OBP2B*: forward, catgggaaagcttgtgggta and reverse, gcgctgcaccaatttctta; *LCN1*: forward, tcagccttggcctcattg and reverse, cagataccacgtccctgaca; *FABP5*: forward, ccacagctgatggcaga and reverse, gacacactccaccact. Table S3 also summarizes the primers used in this study. Results were normalized with the *GAPDH* gene.

#### Cell viability assay

To determine the effects of *trans*-2-nonenal (Toronto Research Chemicals Inc., Toronto, Canada), oleic acid (Nakarai Tesque, Inc., Tokyo, Japan), palmitoleic acid (FUJIFILM Wako Pure Chemical Corporation, Tokyo, Japan), stearic acid (Tokyo Chemical Industry Co., Ltd, Tokyo, Japan), and palmitic acid (Tokyo Chemical Industry Co., Ltd, Tokyo, Japan) on cell viability, keratinocytes were cultured to 50–60% confluency in 24-well culture plates and differentiated for 24h. 1 M stock solutions of *trans*-2-nonenal, oleic acid, palmitoleic acid, and 50 mM stock solutions of stearic acid and palmitic acid were diluted with ethanol and added to wells to give concentrations of 50 μM, 100 μM, 50 μM, 100 μM, 50 μM, respectively. The same amount of ethanol was added to control wells. To prevent loss by evaporation, all wells were sealed with polyolefin micro sealing tape (3 M Japan Limited, Tokyo, Japan) and incubated for 24h. Then, the wells were washed with HBS buffer and the cells were treated with trypsin. After addition of Trypsin Neut Solution (Kurabo Industries Ltd., Osaka, Japan) to stop the enzyme reaction, cells were diluted 1:1 in trypan blue solution (Nacalai tesque Inc., Kyoto, Japan) and viable cells were counted under a microscope. The viability of cells treated with *trans*-2-nonenal, oleic acid, palmitoleic acid, stearic acid, or palmitic acid was calculated as a percentage of that of control cells.

#### siRNA transfection

One day before seeding cells onto 24-well plates for cell viability assay or 12-well Millicells for three-dimensional epidermal equivalent model construction, the cells were grown to 80% confluency, and transfected with 20 nM scramble control, OBP2A siRNA, or FABP5 siRNA (GE Healthcare Dharmacon, Lafayette, CO, USA) using the transfection reagent RNA iMAX (Thermo Fisher Scientific, MA, USA) in OptiMem (Thermo Fisher Scientific, MA, USA) as described in the manual. Scramble control: ugguuuacaugucgacuaa, ugguuuacauguuguguga, ugguuuacauguuuucuga and ugguuuacauguuuuccua. OBP2A siRNA: aggccaugguggucgauaa and cccuggaggaggaggauau or agacggaggagccuggcaa and gcgcuacaugggaaagcuu. FABP5 siRNA: ggagcuaggagugggaaua and guacucggaucuaugaaaa or ggaaauuagugguggagug and ggaguuaauuaagagaaug.

#### Three-dimensional epidermal equivalent model construction

2.2 × 10^5^ keratinocytes/500 mL CnT Prime medium (CELLnTEC, Berne, Switzerland) with or without siRNA (20 nM) were plated on 12-well Millicells with 1.0 mm pore size PET hanging inserts (bore diameter 12 mm, Millipore, Billerica, MA). The inserts were coated with CellStart (Thermo Fisher Scientific, MA, USA) in a 50× dilution of DPBS (Sigma-Aldrich, ST, USA) before cell plating, and 1 mL CnT Prime (CELLnTEC, Bern, Switzerland) was added to each well. 3 days after seeding, the medium was switched to CnT-PR-3D differentiation medium (CELLnTEC, Bern, Switzerland) both inside and outside the inserts. Cultures were submerged in differentiation medium for 24h and then lifted to the air-medium interface by removing the excess medium from the insert and reducing the volume of the differentiation medium on the outside to 500 mL. Cultures were changed daily with 500 mL of differentiation medium for 9 days and then harvested. For evaluation of proliferative cells, BrdU was added to the medium 24h before harvest. IL-4 and IL-13 treatment experiments were performed by adding carrier-free IL-4 and IL-13 (50 ng/mL) to the medium 5 days before harvest.

#### Enzyme-linked immunosorbent assay (ELISA)

Cells were harvested in RIPA buffer (Nacalai tesque Inc., Kyoto, Japan) containing protein inhibitor cocktail (Nacalai tesque Inc., Kyoto, Japan) 1% and homogenized in a glass homogenizer. The lysate was centrifuged (15,000 rpm, 30 min), and protein concentrations were measured with a protein assay kit (BIO-RAD, CA, USA). OBP2A, OBP2B, IL-1α, IL-1β, and IL-6 in the supernatant of cultured medium or the lysate were measured with a Human Odorant Binding Protein 2A (OBP2A) ELISA kit, Human Odorant Binding Protein 2B (OBP2B) ELISA kit (MyBioSource, CA, USA), Human IL-1 alpha/IL-1F1 Quantikine ELISA Kit, Human IL-1 beta/IL-1F2 Quantikine ELISA Kit, or Human IL-6 Quantikine ELISA Kit (R&D Systems, MO, USA) according to the manufacturer’s protocol.

#### Immunoblotting

Samples for immunoblotting were prepared in the same way as for ELISA. Samples were subjected to sodium dodecyl sulfate polyacrylamide gel electrophoresis (Invitrogen, CA, USA) and transferred to PVDF membranes (Invitrogen, CA, USA). Membrane staining was performed by using antibodies to OBP2A (orb582625, Biorbyt Ltd., Cambridge, UK), FABP5 (39926, Cell Signaling Technology, MA, USA), PERK (5683, Cell Signaling Technology, MA, USA), Bip (3177, Cell Signaling Technology, MA, USA), PDI (3501, Cell Signaling Technology, MA, USA), involucrin (ab53112, abcam, Cambridge, UK), keratin 10 (ab76318, abcam, Cambridge, UK), corneodesmosin (13184, proteintech, IL, USA), desmoglein 2 (MAB947, R&D Systems, MO, USA), and β-actin (sc-47778, Santa Cruz Biotechnology, TX, USA) as primary antibodies and a Novex AP Rabbit Chemiluminescent Detection Kit or Novex AP Mouse Chemiluminescent Detection Kit (Invitrogen, CA, USA) as secondary antibodies. The proteins were detected using Chemi-Lumi One Super (Nacalai tesque Inc., Kyoto, Japan). The signal intensity of each sample was measured with ImageJ 1.52a software.

#### Histology

Samples were fixed with 4% paraformaldehyde in PBS, embedded in paraffin, and sectioned at 3 μm for hematoxylin and eosin (H&E) staining and immunostaining. To evaluate the epidermis area, 3 images per section from each condition were taken and the area was measured with ImageJ 1.52a software. BrdU immunostaining was performed with anti-BrdU antibody [BU1/75(ICR1)] (ab6326, Abcam, Cambridge, UK) as the primary antibody and polyclonal biotinylated rabbit anti-rat Ig (#E0468, DAKO, Hovedstaden, Denmark) as the secondary antibody, using ABC (Avidin-Biotin Complex) kits (Vector Laboratories, CA, USA) and DAB substrate (Roche Diagnostics Inc, Basel, Switzerland). Whole images per section were obtained and BrdU-positive cells were counted under a microscope.

For immunostaining of OBP2A, filaggrin, involucrin, keratin 10, transglutaminase 1, claudin 1, corneodesmosin, and desmoglein 2, antibodies to OBP2A (orb37374, Biorbyt Ltd., Cambridge, UK), filaggrin (sc-66192, Santa Cruz Biotechnology, TX, USA), involucrin (ab53112, abcam, Cambridge, UK), keratin 10 (20R-CP002, Fitzgerald Industries International, MA, USA), transglutaminase 1 (sc-166467, Santa Cruz Biotechnology, TX, USA), claudin 1 (71–7800, Invitrogen, CA, USA), corneodesmosin (ab204235, abcam, Cambridge, UK), and desmoglein 2 (MAB947, R&D Systems, MO, USA) were used as primary antibodies, and donkey anti-rabbit IgG (Invitrogen, CA, USA) or donkey anti-guinea pig IgG (Jackson ImmunoResearch, PA, USA) was used as a second antibody. For immunostaining of OBP2A, an iFluor 594 PSA Imaging Kit with goat anti-rabbit IgG (AAT Bioquest, Inc., CA, USA) was used according to the manufacturer’s protocol. Samples were observed with a fluorescence microscope (BX51and DP80, Olympus, Tokyo, Japan) using cellSens software (Olympus, Tokyo, Japan). To evaluate the fluorescence intensity, 3 images per section from each condition were taken and the area was measured with ImageJ 1.52a software.

#### Gravimetric transepidermal water loss (TEWL) measurement

TEWL was measured as follows with reference to previous report.^85^ Inserts of the three-dimensional epidermal equivalent model were placed dermis-side down on silicon rubber plates and the lateral edges were sealed with petrolatum so that water loss occurred only through the epidermal surface. Epidermis model sections were kept at ambient temperature (37°C) and humidity (30–35%) and weighed after 1h. TEWL levels are reported as milligrams of water lost per square millimeter per hour.

#### Measurement of cholesterol and aldehyde

Three-dimensional epidermal equivalent model samples were homogenized in RIPA buffer (Nacalai tesque Inc., Kyoto, Japan) containing protein inhibitor cocktail (Nacalai tesque Inc., Kyoto, Japan) 1% for cholesterol measurement or in HBS containing protein inhibitor cocktail (Nacalai tesque Inc., Kyoto, Japan) 1% for aldehyde measurement. The lysate was centrifuged (15,000 rpm, 30 min), and cholesterol in the supernatant was measured with an Amplite Cholesterol Quantitation Kit (AAT Bioquest, CA, USA) according to the manufacturer’s protocol. Aldehyde in the supernatant was measured with an Aldehyde Assay Kit II (Colorimetric-Blue) (abcam, Cambridge, UK) after filtration of the samples using an Amicon Ultra 3K filter (Millipore, Billerica, MA).

#### LC-MS/MS analysis

Samples for LC-MS/MS analysis were prepared in the same way as described for cholesterol measurement. The lysate was centrifuged (15,000 rpm, 30 min), and total lipids in the supernatant were extracted by the Bligh-Dyer method and mixed with EquiSPLASH (Avanti Polar Lipids Inc., AL, USA) to provide internal standards for quantification by LC-MS/MS analysis. The sample volume for analysis were adjusted so that the amount of protein was the same in all samples. The LC-MS/MS system consisted of a Vanquish UHPLC system (Thermo Fisher Scientific, MA, USA) and Orbitrap Fusion Lumos equipping an electrospray ionization (ESI) source (Thermo Fisher Scientific, MA, USA). All raw files were processed using MS-DIAL (http://prime.psc.riken.jp/compms/msdial/main.html, version 4.48) with MS/MS spectra for identification and relative quantification of lipids. This software is freely available for untargeted metabolomics. The raw MS files (RAW format file) were converted to ABF (analysis base file format) using the freely available Reifycs file converter (http://www.reifycs.com/AbfConverter/) and the ABF files were imported into MS-DIAL. The lipids were quantified as relative values with respect to internal standards contained in EquiSPLASH. The values were obtained as the MS intensity of the target lipid divided by the MS intensity of the appropriate internal standard, which is a stable-isotope-labeled lipid having the same polar head moiety as the target lipid. Since the structure and retention time of the target lipid and internal standard are not exactly the same, we did not calculate the unit such as g or mol. However, the relative values can be compared among the samples because the sample volumes for the analysis were adjusted to achieve the same amount of protein. The lipidomics method was based on a previous report^86^ and the relative quantification method followed the lipidomic standard initiative (https://lipidomics-standards-initiative.org/). All statistical analyses were conducted using MS-DIAL and MetaboAnalyst (https://www.metaboanalyst.ca/, version 6.0), which is freely available software for statistical analysis. Table S1 summarizes lipid compounds detected by LC-MS/MS.

#### ToF-SIMS analysis

3DE-model or skin tissue samples were washed in PBS, soaked in O.C.T. compound (Sakura Finetek Japan Co., Ltd., Tokyo, Japan), immediately frozen in dry ice, and sectioned at 6 μm. The samples were put on glass slides, MALDI MSI (Bruker Daltonics, Bremen, Germany), and kept at −80°C until measurement. To reduce the effect of contamination on the surface of the sections, Ar gas cluster ion beam (Ar-GCIB) etching was performed before the analysis. ToF-SIMS analyses were conducted with TOF.SIMS 5 (IONTOF GmbH, Münster, Germany), using 30 kV Bi_3_^++^ primary ions. Positive and negative ion data were acquired with the instrument optimized for high lateral resolution (lateral resolution ≈500 nm). The identification of free fatty acids in the acquired mass spectra was based on agreement between theoretical mass and observed peak positions in spectra obtained at high mass resolution. Table S2 summarizes theoretical mass and observed peak positions in the analysis.

#### RNA-seq

Three-dimensional epidermal equivalent model samples were homogenized in RLT buffer (QIAGEN, Hilden, Germany) and total RNA was isolated using an RNeasy mini kit (QIAGEN, Hilden, Germany). Transcriptome libraries were constructed by polyA purification. 1 μg of total RNA from each sample was used to construct a cDNA library, followed by sequencing on the DNBSEQ system with pair end 100 bp reads and ∼30 million reads per sample. The quality of FASTQ files was examined using FastQC-0.11.8. The human reference sequence file (NCBI Build 37/hg19) and the GTF annotation file were obtained from iGenomes (http://support.illumina.com/sequencing/sequencing_software/igenome.html). Gene expression analysis was performed with iDEP.95 (http://bioinformatics.sdstate.edu/idep95/). Genes with more than 1 count per million (CPM) and under 0.01 FDR value were considered as significantly differentially expressed genes (DEG).

#### Gene ontology (GO) term enrichment analysis

Functional enrichment was performed in GO terms: Biological process and human gene-disease associations to DEG which has more than 1.5-fold change obtained from RNA-seq with Metascape.^87^ A corrected *p*-value <0.05 was chosen as the threshold for significantly enriched GO terms. In addition, all GO categories have a gene count of 10 or greater.

#### Electron-microscopic observation

Three-dimensional epidermal equivalent model or skin tissue samples for electron microscopy were minced (<0.5 mm^3^ pieces) and fixed overnight in modified Karnovsky’s fixative. They were then post-fixed in 2% aqueous osmium tetroxide or 0.2% ruthenium tetroxide as described previously.^85^ After fixation, all samples were dehydrated in graded ethanol solutions, and embedded in an Epon-epoxy mixture. Stratum corneum/stratum granulosum (SC/SG) lipid domains were quantified using osmium post-fixed material. We used four samples from different subjects for each condition. Measurements were made without knowledge of the prior experimental treatment. The area was measured using photographs of randomly selected sections at a constant magnification with ImageJ 1.52a software.

#### Docking simulation

Three-dimensional structure data of OBP2A (PDB ID: 4RUN) were obtained from RCSB PDB (https://www.rcsb.org/) and chemical structure data of *trans*-2-nonenal, oleic acid, palmitoleic acid, stearic acid, palmitic acid, and urushiol were obtained from PubChem (https://pubchem.ncbi.nlm.nih.gov/). Docking simulation of OBP2A with target molecules was performed with AutoDock Tools (1.5.6) software according to the manual provided by CCSB (https://vina.scripps.edu/)^88^ and the results were visualized with PyMOL (2.1) software.

### Quantification and statistical analysis

The statistical significance of differences among three or more groups was determined by ANOVA with Scheffé's method. Student’s t test was used to determine the significance of differences between two groups. A *p*-value <0.05 was considered significant.

## Supplemental information

Document S1. Figures S1–S6 and Tables S2 and S3

Table S1. Lipid compounds detected by LC-MS/MS
