# Supplementary material for: OBP2A regulates epidermal barrier function and protects against cytotoxic small hydrophobic molecules
Source: iScience. 2024 Oct 2;27(11):111093. doi: 10.1016/j.isci.2024.111093 (PMC11536036; doi:10.1016/j.isci.2024.111093)
Supplement: Document S1. Figures S1–S6 and Tables S2 and S3 [file mmc1.pdf]

iScience, Volume *27*

Supplemental information

OBP2A regulates epidermal barrier function and protects against cytotoxic small hydrophobic molecules

Shinobu Nakanishi, Tatsuya Hasegawa, Katsuyuki Maeno, Akira Motoyama, and Mitsuhiro Denda

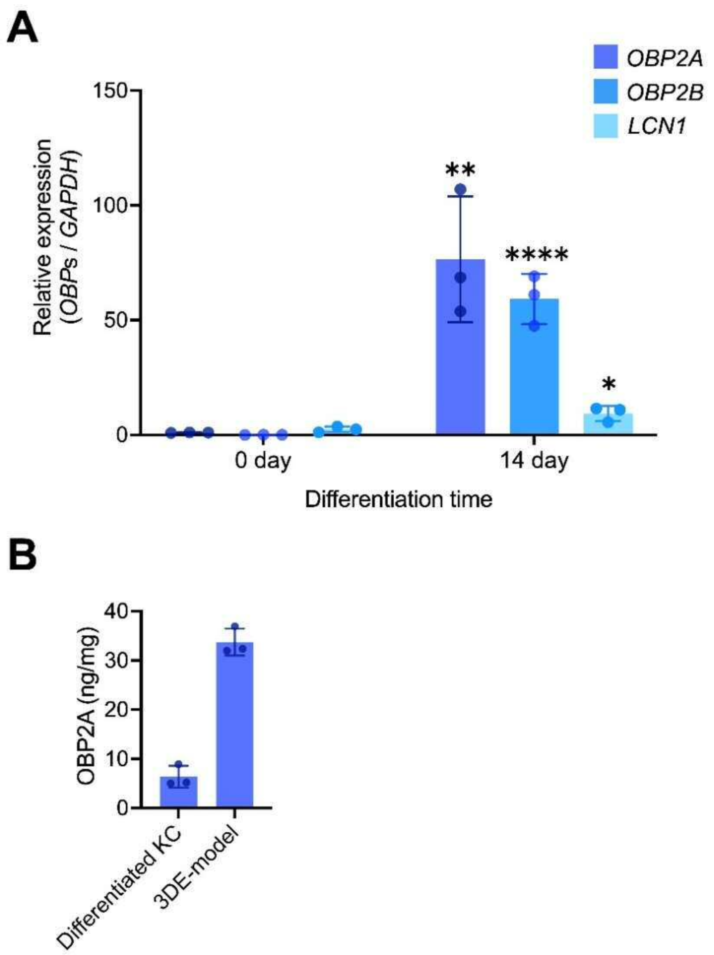

Supplementary figure S1. Expression of OBPs in differentiated keratinocytes (KC) and the 3DE-model, Related to Figure 1.

(A) qPCR analysis of OBPs in the 3DE-model (n = 3). 0 day: before differentiation, 14 day: constructed 3DE-model. (B) ELISA analysis of OBP2A in differentiated keratinocytes and the 3DE-model (n = 3). Bars and lines represent mean ± SD. *: $p < 0.05$, **: $p < 0.01$, ****: $p < 0.0001$ in Student's *t* test.

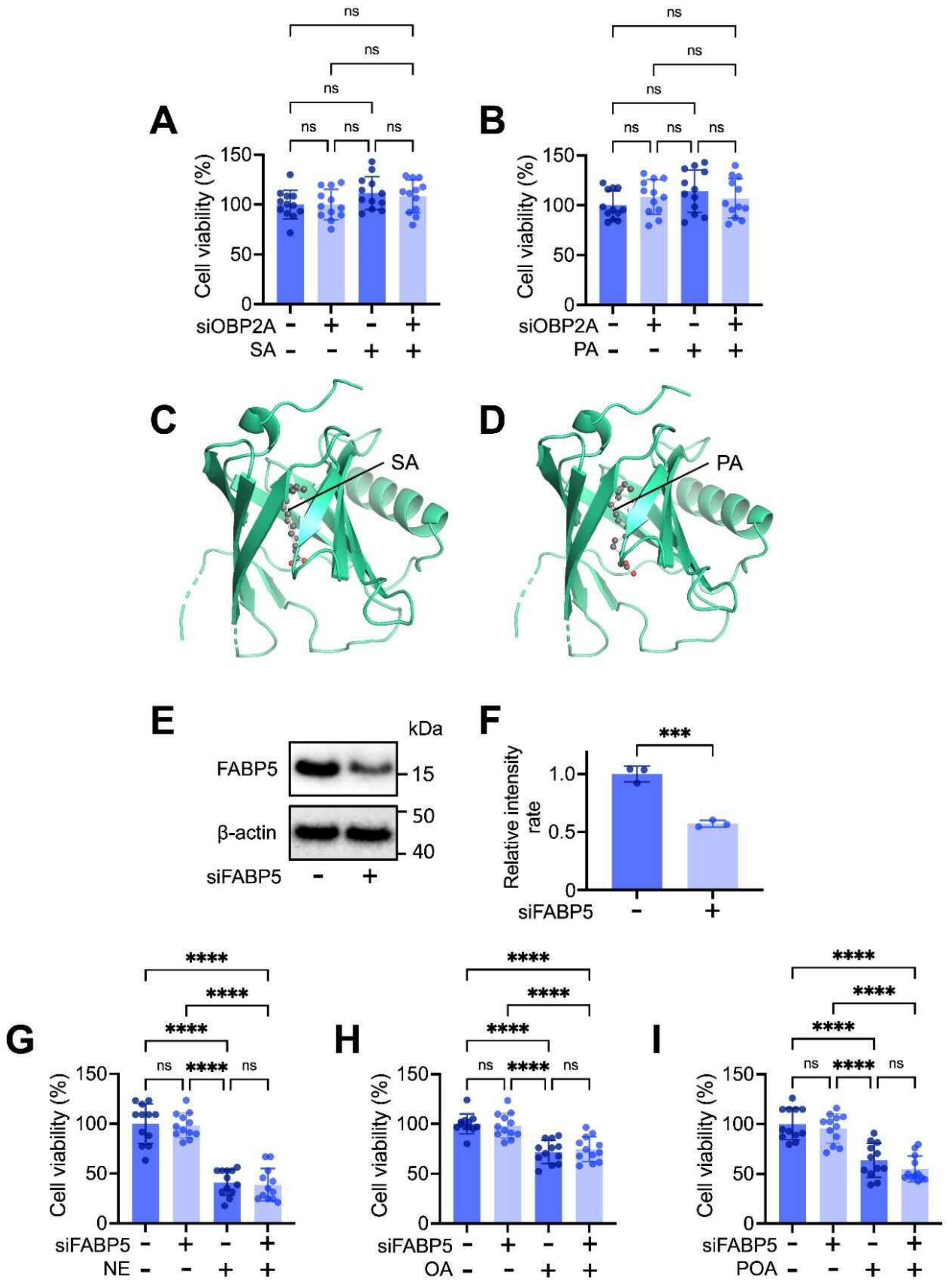

A
B
C
D
E
F
G
H
I
ns
ns
ns
ns
ns
ns
ns
Cell viability (%)
150
100
50
0
siOBP2A - + - +
SA - - + +
PA - - + +
SA
PA
kDa
FABP5
15
β-actin
50
40
siFABP5 - +
***
Relative intensity rate
1.0
0.5
****
siFABP5 - + - +
NE - - + +
OA - - + +
POA - - + +

Internal

Supplementary figure S2. Effects of OBP2A and FABP5 on the viability of cells treated with stearic acid, palmitic acid, *trans*-2-nonenal, oleic acid, or palmitoleic acid, Related to Figure 2.

(A) (B) Viability of keratinocytes treated with stearic acid (SA) or palmitic acid (PA) (n = 12). The cells were treated with scrambled siRNA or OBP2A siRNA. Anova F value = 1.691, p = 0.1827 for SA, Anova F value = 1.197, p = 0.3219 for PA. (C) (D) Docking simulation of SA, or PA to OBP2A. Gray: carbon atom, Red: oxygen atom, green: OBP2A (PDB ID: 4RUN). (E) Western-blot analysis for the detection of FABP5 in cells. (F) Relative value of FABP5 western-blot signal (n = 3). (G) (H) (I) Viability of keratinocytes treated with *trans*-2-nonenal (NE), oleic acid (OA), or palmitoleic acid (POA) (n = 12). The cells were treated with scrambled siRNA or FABP5 siRNA. Anova F value = 56.82, $p < 0.0001$ for NE, Anova F value = 19.66, $p < 0.0001$ for OA, Anova F value = 26.39, $p < 0.0001$ for POA. Bars and lines represent mean ± SD. ***: $p < 0.0005$, ****: $p < 0.0001$, ns: not significant in ANOVA with Scheffé's method (A) (B) (G) (H) (I) and Student’s t test (F).

Internal

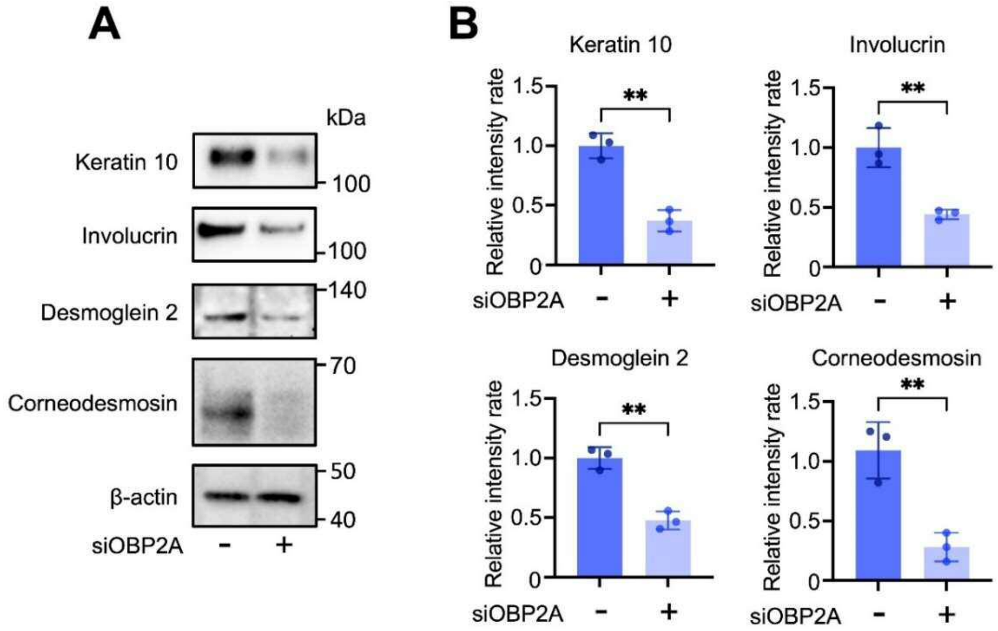

Supplementary figure S3. Reduction of epidermal barrier-related proteins and desmosomal proteins in the OBP2A-knockdown 3DE-model, Related to Figure 3 and Figure 4.

(A) Western-blot analysis for the detection of epidermal barrier-related proteins and desmosomal proteins in the OPB2A-knockdown 3DE-model. (B) Relative values of western-blot signal of epidermal barrier-related proteins and desmosomal proteins in the 3DE-model (n = 3). Bars and lines represent mean ± SD. **: $p < 0.01$ in Student's t test.

Internal

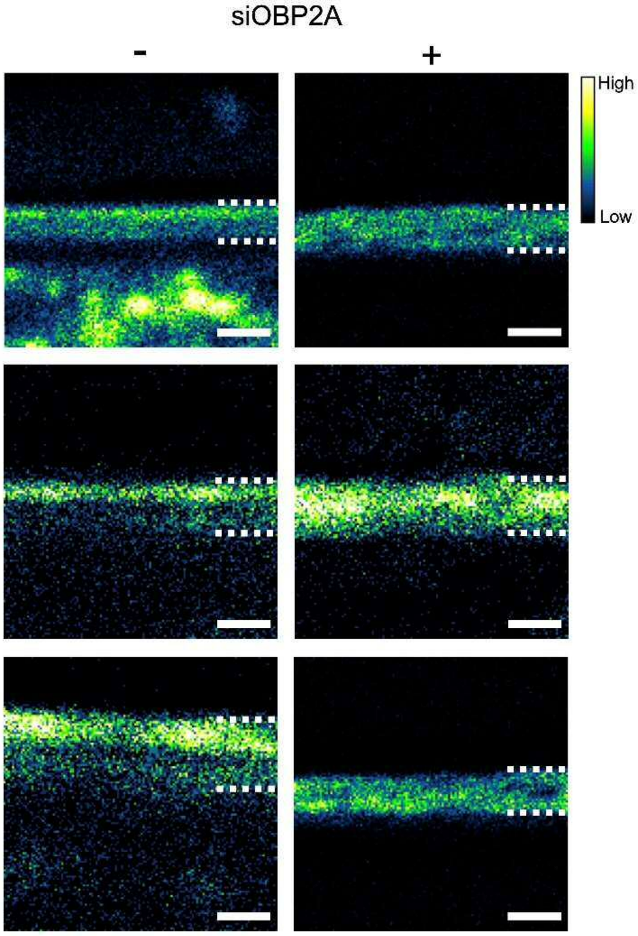

Supplementary figure S4. Influence of OBP2A-knockdown on distribution of free fatty acid, Related to Figure 5.

(A) Image of total free fatty acid in the 3DE-model obtained with ToF-SIMS. n = 3. Bars = 100 μm.

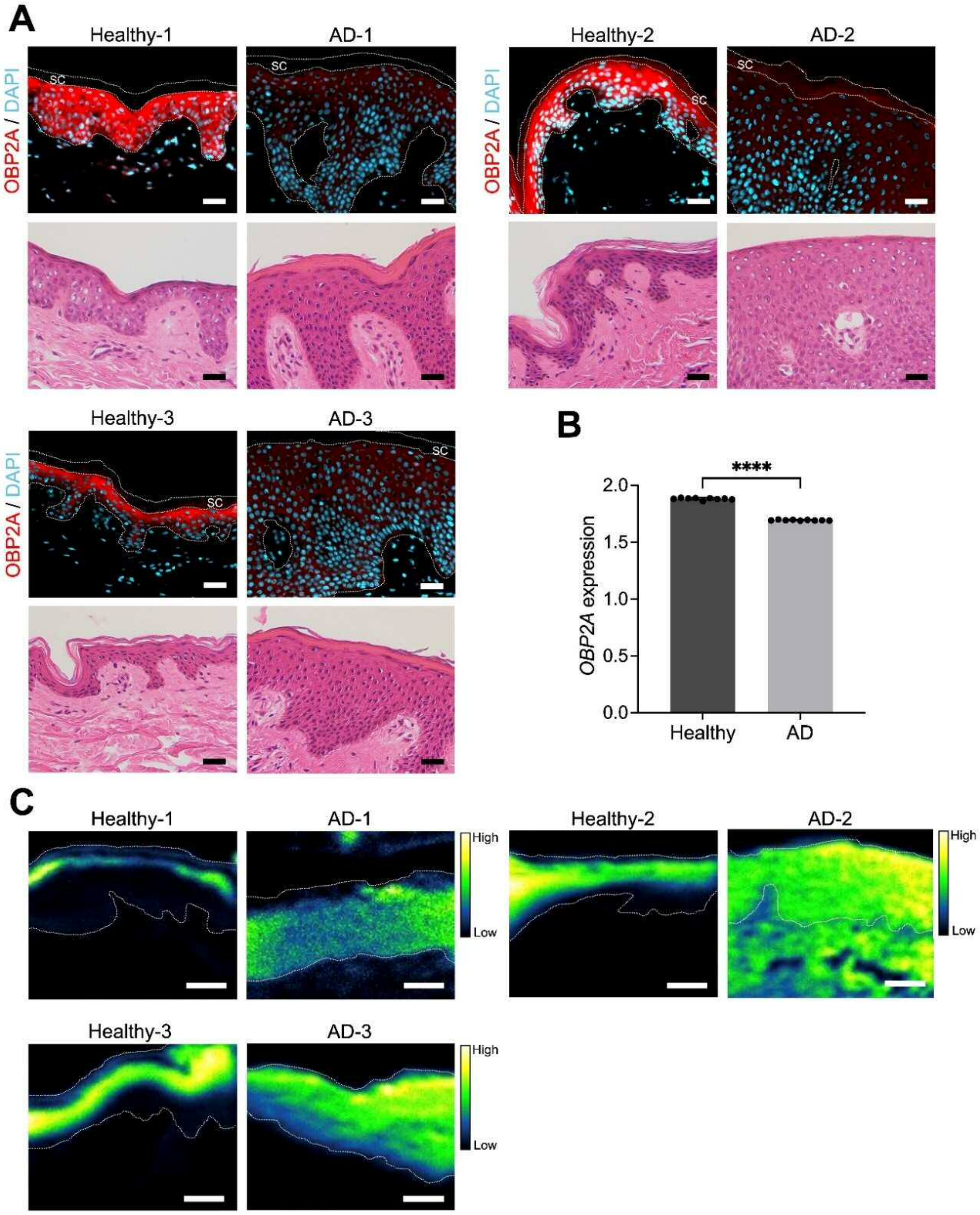

Supplementary figure S5. Expression of OBP2A and image of total free fatty acid obtained with ToF-SIMS in healthy or atopic dermatitis lesional skin, Related to Figure 6.

(A) H&E staining and immunofluorescence staining of OBP2A (3 donors). SC: stratum corneum. Bars = 30 µm. (B) OBP2A expression in publicly available DNA microarray data of atopic dermatitis lesional skin compared with healthy skin (GSE16161). (C) Image of total free fatty acid obtained with ToF-SIMS (3 donors). AD: atopic dermatitis. Bars = 100 µm. Bars and lines represent mean ± SD. ****: $p < 0.0001$ in Student's *t* test.

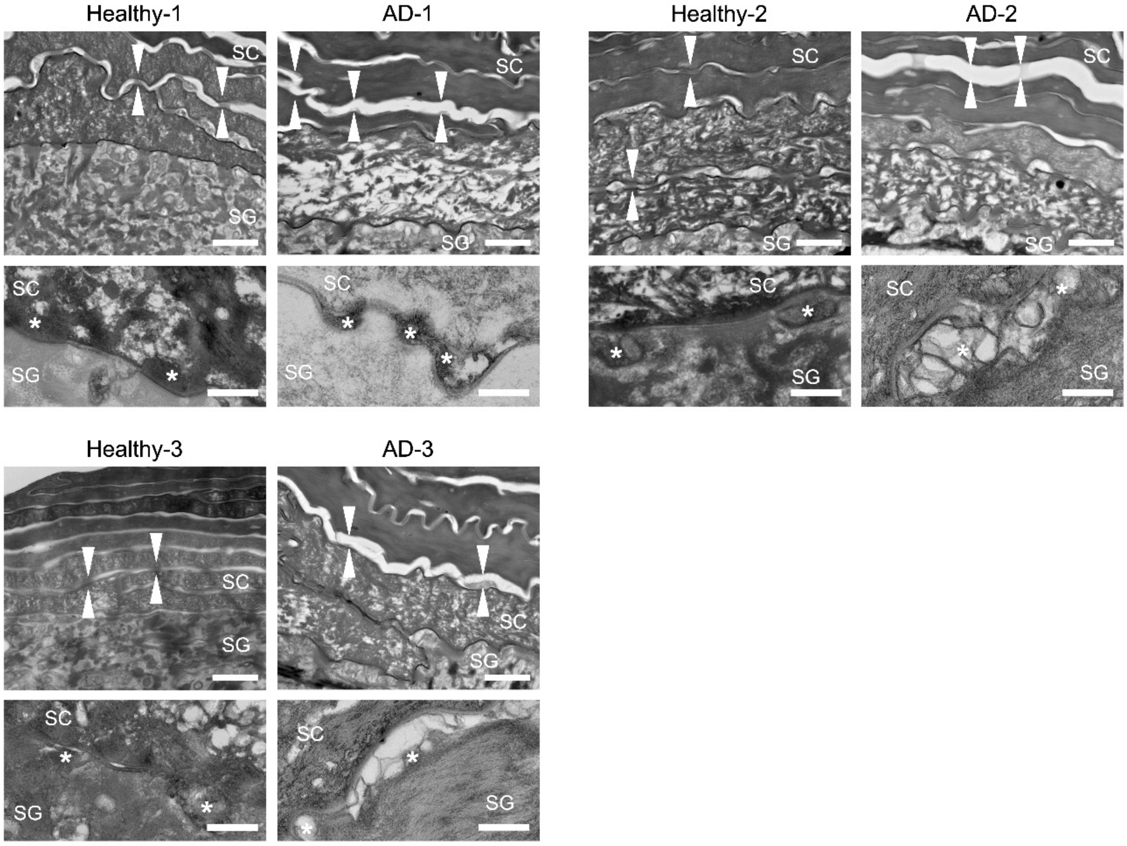

Supplementary figure S6. Electron-microscopic images of healthy skin and atopic dermatitis lesional skin, Related to Figure 6.

Upper: osmium staining, Bars = 1 µm. Lower: ruthenium staining, Bars = 400 nm (3 donors). AD: atopic dermatitis, SG: stratum granulosum, SC: stratum corneum, white arrows: corneodesmosomes in the SC, *: intercellular lipid domains.

Table S2. The identification of free fatty acids based on the agreement between theoretical masses and observed peaks in the Tof-SIMS analysis.

Formula	*m/z*	
	observed	theoretical
O^-	16.00	15.99
Cl^-	34.97	34.97
CNO^-	42.00	42.00
PO_3^-	78.96	78.96
$PO_4H_2^-$	96.97	96.97
$C_4H_4N_3O^-$	110.03	110.04
$C_4H_3N_2O_2^-$	111.02	111.02
$C_5H_5N_2O_2^-$	125.04	125.04
$C_5H_4N_5^-$	134.05	134.05
$C_5H_4N_5O^-$	150.04	150.04
$C_{12}H_{21}O_2^-$	197.15	197.15
$C_{12}H_{23}O_2^-$	199.17	199.17
$C_{14}H_{25}O_2^-$	225.19	225.19
$C_{14}H_{27}O_2^-$	227.20	227.20
$C_{16}H_{27}O_2^-$	251.20	251.20
$C_{16}H_{29}O_2^-$	253.22	253.22
$C_{16}H_{31}O_2^-$	255.23	255.23
$C_{18}H_{29}O_2^-$	277.22	277.22
$C_{18}H_{31}O_2^-$	279.23	279.23
$C_{18}H_{33}O_2^-$	281.25	281.25
$C_{18}H_{35}O_2^-$	283.26	283.26
$C_{20}H_{31}O_2^-$	303.24	303.23
$C_{20}H_{33}O_2^-$	305.25	305.25
$C_{20}H_{35}O_2^-$	307.26	307.26
$C_{20}H_{37}O_2^-$	309.28	309.28
$C_{27}H_{45}O^-$	385.34	385.35
$C_{29}H_{49}O_2^-$	429.38	429.37

Internal

Table S3. Primers used in this study.

Primers	Forward	Reverse
GAPDH	gaaggtgaaggtcggagtc	gaagattggtgatgggatttc
OBP2A	gagcctggcaaattcagc	tctttgcagtaaaagacgtagtcg
OBP2B	catgggaaagcttgtgggta	gcgctgcaccaatttctta
LCN1	tcagccttggcctcattg	cagataccacgtccctgaca
FABP5	ccacagctgatggcaga	gacacactccaccact

Internal
